# Efficacy of non-invasive and invasive respiratory management strategies in adult patients with acute hypoxaemic respiratory failure: a systematic review and network meta-analysis

**DOI:** 10.1186/s13054-021-03835-8

**Published:** 2021-11-29

**Authors:** Masaaki Sakuraya, Hiromu Okano, Tomoyuki Masuyama, Shunsuke Kimata, Satoshi Hokari

**Affiliations:** 1 Department of Emergency and Intensive Care Medicine, JA Hiroshima General Hospital, Jigozen 1-3-3, Hatsukaichi, Hiroshima 738-8503 Japan; 2 Department of Critical and Emergency Medicine, National Hospital Organization Yokohama Medical, Yokohama, Japan; 3 Department of Emergency and Critical Care Medicine, Misato Kenwa Hospital, Saitama, Japan; 4 Department of Preventive Services, School of Public Health, Kyoto University, Kyoto, Japan; 5 Department of Respiratory Medicine and Infectious Diseases, Niigata University Graduate School of Medical and Dental Sciences, Niigata, Japan

**Keywords:** Acute hypoxaemic respiratory failure, Continuous positive airway pressure, High-flow nasal oxygen, Network meta-analysis, Non-invasive ventilation

## Abstract

**Background:**

Although non-invasive respiratory management strategies have been implemented to avoid intubation, patients with de novo acute hypoxaemic respiratory failure (AHRF) are high risk of treatment failure. In the previous meta-analyses, the effect of non-invasive ventilation was not evaluated according to ventilation modes in those patients. Furthermore, no meta-analyses comparing non-invasive respiratory management strategies with invasive mechanical ventilation (IMV) have been reported. We performed a network meta-analysis to compare the efficacy of non-invasive ventilation according to ventilation modes with high-flow nasal oxygen (HFNO), standard oxygen therapy (SOT), and IMV in adult patients with AHRF.

**Methods:**

The Cochrane Central Register of Controlled Trials, MEDLINE, EMBASE, and Ichushi databases were searched. Studies including adults with AHRF and randomized controlled trials (RCTs) comparing two different respiratory management strategies (continuous positive airway pressure (CPAP), pressure support ventilation (PSV), HFNO, SOT, or IMV) were reviewed.

**Results:**

We included 25 RCTs (3,302 participants: 27 comparisons). Using SOT as the reference, CPAP (risk ratio [RR] 0.55; 95% confidence interval [CI] 0.31–0.95; very low certainty) was associated significantly with a lower risk of mortality. Compared with SOT, PSV (RR 0.81; 95% CI 0.62–1.06; low certainty) and HFNO (RR 0.90; 95% CI 0.65–1.25; very low certainty) were not associated with a significantly lower risk of mortality. Compared with IMV, no non-invasive respiratory management was associated with a significantly lower risk of mortality, although all certainties of evidence were very low. The probability of being best in reducing short-term mortality among all possible interventions was higher for CPAP, followed by PSV and HFNO; IMV and SOT were tied for the worst (surface under the cumulative ranking curve value: 93.2, 65.0, 44.1, 23.9, and 23.9, respectively).

**Conclusions:**

When performing non-invasive ventilation among patients with de novo AHRF, it is important to avoid excessive tidal volume and lung injury. Although pressure support is needed for some of these patients, it should be applied with caution because this may lead to excessive tidal volume and lung injury.

*Trial registration* protocols.io (Protocol integer ID 49375, April 23, 2021). 10.17504/protocols.io.buf7ntrn.

**Supplementary Information:**

The online version contains supplementary material available at 10.1186/s13054-021-03835-8.

## Background

Acute hypoxaemic respiratory failure (AHRF) is the most common cause of intensive care unit (ICU) admission among adult patients, with a hospital mortality rate of approximately 30% [1]. Non-invasive respiratory management has been investigated widely among patients with AHRF. Non-invasive ventilation is recommended to reduce the risk of endotracheal intubation and mortality in patients with AHRF, especially due to cardiopulmonary oedema [2]. Compared with standard oxygen therapy (SOT), high-flow nasal oxygen (HFNO) is also a preferable option for patients with AHRF [3].

While non-invasive ventilation has been reported to be used in 15% of patients with acute respiratory distress syndrome (ARDS), it may be associated with higher ICU mortality, especially in patients with severe hypoxaemia [4]. A definite diagnosis of ARDS [5] may be difficult or impossible before the implementation of respiratory management strategies, because the precise measurement of the actual inspired fraction of oxygen may be unavailable and the positive end-expiratory pressure (PEEP) not used. Furthermore, when implementing non-invasive respiratory management strategies in patients with AHRF, we need to consider the cause of the respiratory failure, especially whether it was an established disease for efficacy of non-invasive ventilation including cardiopulmonary oedema or not. De novo AHRF refers to AHRF that occurs without any prior chronic respiratory diseases [6]. Most patients in this category have pneumonia or ARDS with neither heart failure nor chronic obstructive pulmonary disease (COPD). Non-invasive ventilation is not recommended in patients with de novo AHRF [6], and the efficacy of the HFNO has not been consistent among these patients [7, 8].

Excessive tidal volume has been reported to be associated with treatment failure in patients with AHRF [9], and treatment failure has been shown to increase hospital mortality [4]. Although pressure support is needed for hypercapnic respiratory failure, the role of pressure support is unclear in patients with de novo AHRF. Furthermore, there may the possibility of increasing tidal volume and lung injury. A systematic review and network meta-analysis (NMA) was performed recently to evaluate the efficacy of non-invasive respiratory management strategies in adult patients with AHRF, compared with SOT [10]. This NMA divided non-invasive ventilation into two categories: those using a face mask and those using a helmet interface, and showed that helmet non-invasive ventilation was the most effective method to reduce the risk of all-cause mortality and endotracheal intubation. Moreover, continuous positive airway pressure (CPAP) was used as a non-invasive ventilation mode along with helmet non-invasive ventilation in most randomized controlled trials (RCTs) included in this NMA. However, no meta-analyses have evaluated the efficacy of non-invasive ventilation according ventilation mode among patients with AHRF. Furthermore, in the previous NMA, non-invasive respiratory management strategies were not compared with invasive mechanical ventilation (IMV). Although non-invasive respiratory management strategies have been used to avoid complications of IMV and improve clinical outcomes, few meta-analyses comparing non-invasive respiratory management strategies with IMV have been reported.

When performing non-invasive ventilation in patients with AHRF, both PEEP and pressure support are expected to improve oxygenation. However, tidal recruitment provided by pressure support may contribute to not only oxygenation improvement but also lung injury. In this study, we hypothesized that CPAP was the most effective strategy for reducing mortality and endotracheal intubation in patients with de novo AHRF. We performed an NMA to compare the efficacy of non-invasive ventilation according to the ventilation modes with HFNO, SOT, and IMV in adult patients with AHRF.

## Methods

### Protocol and registration

This systematic review was designed according to the Preferred Reporting Items for Systematic review and Meta-Analyses extension statement for reviews incorporating network meta-analyses (details shown in Additional file 1: Table S1) [11], and the protocol was registered at protocols.io (Protocol integer ID 49,375) [12].

### Eligibility criteria

#### Type of studies

We included all the RCTs reported in the publication status (published, unpublished, and academic abstracts). Randomized crossover, cluster-randomized, or quasi-experimental trials were excluded.

#### Type of participants

This review included adults (age ≥ 18 years) with AHRF, defined by any of the following criteria: new onset (< 7 days) of clinical signs (e.g. tachypnoea, increased work of breathing); radiologic signs (unilateral or bilateral chest X-ray opacities); and hypoxaemia. Hypoxaemia was defined as the ratio of arterial oxygen partial pressure to fractional inspired oxygen (P/F ratio) below 300 cmH_2_O, arterial or percutaneous oxygen saturation < 94% in room air, or partial pressure of arterial oxygen < 60 mmHg in room air or < 80 mmHg with oxygen. The current meta-analysis excluded studies in which more than half of the patients in whom there was the presence of cardiopulmonary oedema, acute exacerbation of COPD or acute exacerbation of asthma, hypercapnia (e.g. > 50 mmHg), post-extubation respiratory failure, post-surgical status, trauma, do-not-resuscitate orders, or limited intervention in the emergency department or pre-hospital care. The rationale for excluding studies that had a primary enrolment of these patients was based on the established efficacy of non-invasive ventilation [6, 13], the possibility of increasing non-pulmonary causes (e.g. airway problem, atelectasis due to pain or surgical procedures, and chest wall instability), and uncertain effects due to limited resources.

#### Types of interventions and comparators

We included RCTs that compared at least two of the following five methods:

1. SOT: Low-flow nasal cannula, face mask, and venturi mask (with no limit on the flow rate).
2. CPAP: CPAP was used as an initial non-invasive ventilation mode. The type of interface, duration of ventilation, management during the non‐invasive ventilation interval, and methods of weaning were not limited.
3. PSV: Pressure support ventilation (PSV), pressure control ventilation, bi-level positive airway pressure, or spontaneous/timed were used initial non-invasive ventilation modes. The type of interface, duration of ventilation, management during the non‐invasive ventilation interval, and methods of weaning were not limited.
4. HFNO: The flow rates and fractions of inspired oxygen were not limited.
5. IMV: Mechanical ventilation via endotracheal intubation not tracheostomy with or without a lung-protective strategy.

#### Type of outcomes

The primary outcomes were short-term mortality measured at the longest time point reported in the follow-up period (< 100 days), ICU discharge, and hospital discharge. The secondary outcome was the incidence of intubation during the ICU stay.

### Information sources

We searched the following databases for eligible trials: The Cochrane Central Register of Controlled Trials; MEDLINE via PubMed; EMBASE; and Ichushi, a database of Japanese research papers. Using a manual search, we also included studies and all systematic reviews on clinical questions about non-invasive respiratory management strategies in the Japanese ARDS Clinical Practice guideline.

### Search

We used the terms ‘ARDS’, ‘adult respiratory distress syndrome’, ‘respiratory failure’, or ‘acute lung injury’ AND ‘non-invasive ventilation’, ‘NIV’, ‘oxygen therapy’, ‘HFNO’, or ‘high-flow therapy’ in searches performed in June 2020 (details in Additional file 1: Table S2). A literature search was also performed from the inception of the database up to May 30, 2021. Search terms included ‘pediatric’ or ‘neonate’ and we included articles written only in English and Japanese, because the systematic review was performed originally for clinical questions in the Japanese ARDS Clinical Practice guideline for adults and pediatrics. During the screening process, we excluded studies with pediatric patients.

### Study selection

Two of the five physicians (HO, TM, SH, SK, and MS) screened the title and abstract or the full text for relevant studies during the first and second screenings, respectively, and extracted data from the included studies into standardized data forms, independently. Disagreements, if any, were resolved through discussions with one of five physicians who did not screen that particular study; the original authors were contacted for clarification, as required. For abstract-only studies that could not be evaluated for eligibility based on our review criteria, we attempted to contact the authors. Discrepancies between two reviewers were resolved through mutual discussions or discussions with a third reviewer, as needed.

### Data collection process

After identifying studies in the second screening, data were extracted from each study by the reviewers (HO, TM, SH, SK, and MS) using two tools: the Cochrane Data Collection Form (RCTs only) [14] and Review Manager software (RevMan version 5.4.1, The Cochrane Collaboration, 2020) [15]. For cases with unknown data, the authors were contacted.

### Data items

We extracted the following study characteristics:

1. Methods: study design, total study duration, number and locations of study centres, study setting, withdrawals, date of study initiation, and funding sources.
2. Participants: number, mean age, age range, sex, severity of condition, diagnostic criteria, and inclusion/exclusion criteria.
3. Interventions: treatment approaches and comparison methods.
4. Outcomes: primary and secondary outcomes that were specified and collected and the timepoints reported.

### Geometry of the network

Network plots were constructed to determine the number of studies and patients included in this meta-analysis. We demonstrated the network geometry that presented the nodes as interventions and each head-to-head direct comparison as lines connecting these nodes. The size of the nodes was proportional to the number of participants in each node. The thickness of the connecting line was proportional to the number of randomized clinical trials in each comparison.

### Risk of bias within individual studies

The risk of bias of outcomes in the included studies was assessed independently by two of the five authors (HO, TM, SH, SK, and MS) using a modified version of the Cochrane ‘Risk of Bias’ instrument [16]. They assessed the overall risk of bias as the worst in any of the following domains: from the randomisation process, deviations from intended interventions, missing outcome data, measurement of the outcomes, and selection of the reported results. The risk of each bias was graded as ‘low risk of bias’, ‘some concerns’, or ‘high risk of bias’. Discrepancies between two reviewers were resolved through discussion among themselves or with a third reviewer, as necessary.

### Planned methods of analyses

#### Direct comparison meta-analysis

A pair-wise meta-analysis was performed using RevMan 5.3 (RevMan 2014) [15]. Forest plots were used for the meta-analysis, and the effect size was expressed as risk ratio (RRs) with 95% confidence intervals (CIs) for the categorical data. The outcome measures were pooled using a random-effects model for the measure of study-specific effects. For all the analyses, a two-sided *P*  < 0.05 was considered to be statistically significant.

#### Network comparison meta-analysis

##### Data synthesis

An NMA was performed using a frequentist approach with multivariate random-effects meta-analysis with the mvmeta command in Stata 15.1 (StataCorp LLC, College Station, TX, USA).

The network meta command allowed us to fit consistency models and estimate network RRs for each treatment strategy based on both direct and indirect comparisons [17]. We constructed forest plots of the RRs with 95% CIs for each treatment strategy in the network.

##### Ranking

Ranking plots (rankograms) were constructed based on the probability that a given treatment had the highest event rate for each outcome. The surface under the cumulative ranking curve (SUCRA), which is a simple transformation of the mean rank, was used to determine the treatment hierarchy [18]. Higher values of the SUCRA statistic, which range from 0 to 100%, increase the likelihood that a therapy is ranked among the best in an NMA [19].

### Assessment of inconsistency

Study heterogeneity among trials for each outcome was assessed by inspecting the forest plots visually and using the I^2^ statistic to quantify any inconsistencies [20]. Publication bias was assessed visually using a funnel plot [19].

Coherence in NMA referred to consistency in the estimates of treatment effects between direct and indirect comparisons [21]. For each pair-wise comparison, we assessed the coherence using a node-splitting method [22]. We also examined coherence globally across the network using the Wald Chi-square test, obtained by fitting the inconsistency model [17].

### Grades of Recommendation, Assessment, Development and Evaluation Working Group (GRADE) assessments of the certainty of evidence for each network comparison

To assess the certainty of evidence for direct comparisons, we used a standard GRADE methodology [23–25]. We rated down for risk of bias, indirectness, inconsistency, and publication bias but did not rate down for imprecision because this occurred at a later step [26, 27]. For indirect comparisons, we started with the lowest certainty of evidence for the contributing direct comparisons and then rated down if there was substantial intransitivity. The transitivity assumption underlying the NMA was evaluated by comparing the distribution of clinical and methodological variables that could act as effect modifiers across treatment comparisons. We assessed the certainty in each network comparison considering the highest certainty of evidence between the direct and indirect evidence [28]; the network estimate was rated subsequently taking into account the imprecision and incoherence [29, 30].

### Additional analysis

A pre-planned sensitivity analysis, which excluded studies using a helmet interface, was performed to assess the robustness of the findings. In addition, we performed post hoc sensitivity analyses to explore the sources of significant incoherence that were present for the primary outcome. The post hoc sensitivity analyses were performed as follows: according to oxygenation (mean P/F ratio > 150 or ≤ 150) and immunocompromised status; excluding studies that enrolled any patients with COPD or cardiopulmonary oedema and studies with high risk of bias; and including studies that reported short-term mortality within 30 days and studies published after 2000.

## Results

### Study selection

**Fig. 1:**
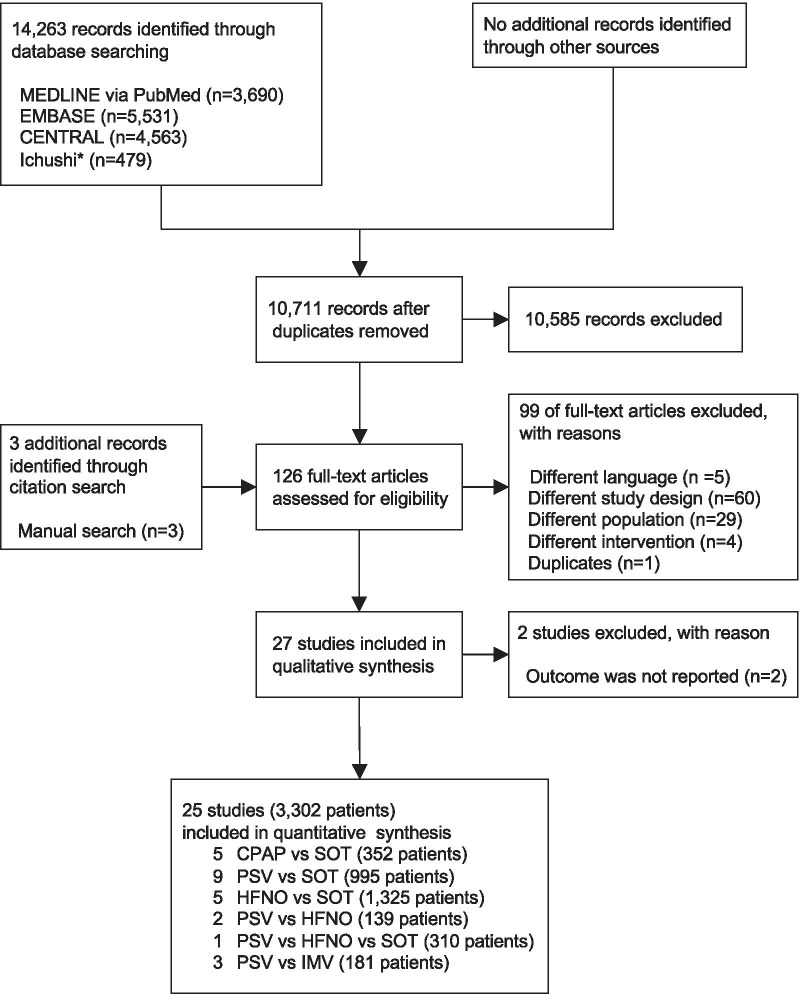
Flow diagram of studies included in this review. *Ichushi is a database of Japanese research papers. CENTRAL, Cochrane Central Register of Controlled Trials; CPAP, continuous positive airway pressure; HFNO, high-flow nasal oxygen; IMV, invasive mechanical ventilation; PSV, pressure support ventilation; RCT, randomized controlled trial; RR, risk ratio; SOT, standard oxygen therapy

The search strategy identified 14,263 records, including 25 RCTs (3302 participants; range 30–776 participants) that were eligible for inclusion (Fig. 1).

### Presentation of network structure and summary of network geometry

**Fig. 2:**
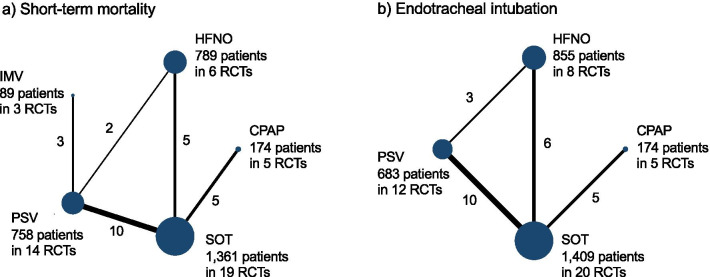
Network plot for non-invasive respiratory management strategies for adults with AHRF. **a** For the primary outcome, short-term mortality, the longest follow-up was up to 100 days. **b** Secondary outcome, endotracheal intubation. When RCTs for direct comparisons exist, this is shown by connections between nodes. The size of the node represents the number of participants who received the intervention. The thickness of lines connecting nodes represents the number of trials for that comparison. CPAP, continuous positive airway pressure; HFNO, high-flow nasal oxygen; IMV, invasive mechanical ventilation; PSV, pressure support ventilation; RCT, randomized controlled trial; SOT, standard oxygen therapy

The included trials evaluated five different interventions, and these included five of 10 potential head-to-head comparisons for short-term mortality as well as four different interventions and four of six potential head-to-head comparisons for intubation. Specifically, nine trials compared PSV with SOT [31–39], five trials compared CPAP with SOT [40–44], five trials compared HFNO with SOT [8, 45–48], three trials compared PSV with IMV [49–51], and two trials compared PSV with HFNO [52, 53] (Table 1; Fig. 2). In addition, a three-group study directly compared PSV with HFNO and SOT [7]. No studies compared CPAP or HFNO with IMV. There were 27 comparisons in 25 RCTs.

**Table 1:** Summary of the characteristics of the studies included in the network meta-analysis

Source	Funding	Total No. of patients	Main cause for respiratory failure	No. of immunocompromised patients, n (%)	Age, year	P/F ratio	RR, /min	PaCO_2_, mmHg	Main exposure	Interface, NIV mode	Comparator	Outcomes of interest assessed	Timing of measurement for mortality
Wysock [31]	Undisclosed	41	Mixed ARF (CAP 39.0%, CPO 34.1%)	NA	63	207	35	43	Non-invasive ventilation (*N* = 21)	Face mask, pressure support	Standard oxygen (*N* = 20)	Mortality, intubation	ICU discharge
Antolnelli [49]	Undisclosed	64	Mixed ARF (ARDS 25.0%, atelectasis 25.0%, CPO 18.8%)	0 (0)	54	120	39	40	Non-invasive ventilation (* N* = 32)	Face mask, pressure support	Invasive ventilation, tidal volume 10 ml/kg (*N* = 32)	Mortality	Hospital discharge
Confalonieri [32]	Undisclosed	56	CAP	NA	64	175	37	49	Non-invasive ventilation (* N* = 28)	Face mask, pressure support	Standard oxygen (*N* = 28)	Mortality, intubation	2-month
Antonelli [33]	Undisclosed	40	Mixed ARF (ARDS 37.5%, atelectasis 25.0%, CPO 22.5%)	40 (100)	67	NA	NA	40	Non-invasive ventilation (*N* = 20)	Face mask, pressure support	Standard oxygen (*N* = 20)	Mortality, intubation	Hospital discharge
Delcaux [40]	Vital Signs Inc	123	Mixed ARF (CAP 42.3%)^a^	NA	58^b^	144^a^	33	36^b^	Non-invasive ventilation (*N* = 62)	FACE mask, CPAP	Standard oxygen (*N* = 61)	Mortality, intubation	Hospital discharge
Martin [41]	Undisclosed	61	Mixed ARF (non-COPD disease 62.3%)	NA	61	199	28	57	Non-invasive ventilation (*N* = 32)	Nasal mask, CPAP	Standard oxygen (*N* = 29)	Mortality, intubation	ICU discharge
Hilbert [34]	Undisclosed	52	CAP	52 (100)	49	139	36	38	Non-invasive ventilation (*N* = 26)	Face mask, pressure support	Standard oxygen (*N* = 26)	Mortality, intubation	Hospital discharge
Ferrer [35]	Red GIRA, Red Respira, and Carburos Metalicos SA	105	Mixed ARF (CAP 32.4%, CPO 28.6%, ARDS 14.3%)	19 (18.1)	62	103	37	37	Non-invasive ventilation (*N* = 51)	Face mask, pressure support	Standard oxygen (*N* = 54)	Mortality, intubation	ICU discharge
Cosentini [42]	Undisclosed	47	CAP^c^	0 (0)	69	248	27	35	Non-invasive ventilation (*N* = 20)	Helmet, CPAP	Standard oxygen (*N* = 27)	Mortality, intubation	Hospital discharge
Squadrone [43]	Regione Piemonte (CEP AN RAN 07) and Ministero dell’Università (PRIN RANI 07)	40	Mixed ARF^c^	40 (100)	49	269	30	36	Non-invasive ventilation (*N* = 20)	Helmet, CPAP	Standard oxygen (*N* = 20)	Mortality, intubation	Hospital discharge
Wermke [36]	Undisclosed	86	CAP^c^	86 (100)	52^b^	270	NA	NA	Non-invasive ventilation (*N* = 42)	Face mask, pressure support	Standard oxygen (*N* = 44)	Mortality, intubation	100 days
Zhan [37]	Beijing Municipal Science and Technology Commission Program	40	ALI	11 (27.5)	46	230	20	32	Non-invasive ventilation (*N* = 21)	Face mask, pressure support	Standard oxygen (*N* = 19)	Mortality, intubation	Hospital discharge
Brambilila [44]	RCCS Fondazione Ca’Granda, Ospedale Maggiore Policlinico, Milan	81	CAP^c^	26 (32.1)	67	141	34	33	Non-invasive ventilation (*N* = 40)	Helmet, CPAP	Standard oxygen (*N* = 41)	Mortality, intubation	Hospital discharge
Azevedo [52]	Undisclosed	30	Mixed ARF (CPO 43.3%, CAP 33.3%)	NA	67	NA	NA	NA	Non-invasive ventilation (*N* = 16)	Face mask, pressure support	High-flow nasal oxygen (*N* = 14)	Intubation	ICU discharge
Frat [7]	French Ministry of Health	310	Mixed ARF (CAP 63.5%)^c^	82 (26.5)	60	155	33	35	Non-invasive ventilation (*N* = 110)	Face mask, pressure suppose	High-flow nasal oxygen (*N* = 106); standard oxygen (*N* = 94)	Mortality, intubation	90 days
Lamiale [45]	Fisher & Paykel	100	Mixed ARF (sepsis related 50%, CPO 7.0%)	100 (100)	62	114	27	NA	High-flow nasal oxygen (*N* = 52)	–	Standard oxygen (*N* = 48)	Intubation	ICU discharge
Lemiale [38]	Legs Poix (Chancellerie des Universités de Paris) and OUTCOMEREA Study Group	374	Mixed ARF (Pneumonia 68.7%)^c^	374 (100)	63^b^	142	26	NA	Non-invasive ventilation (*N* = 191)	Face mask, pressure support	Standard oxygen (*N* = 183)	Mortality, intubation	28 days
Jones [46]	Greenlane Research and Education Fund	303	Mixed ARF (COPD 23.9%, Pneumonia 23.8%, CPO 14.2%)	NA	73	NA	33	NA	High-flow nasal oxygen (*N* = 165)	–	Standard oxygen (*N* = 138)	Mortality, intubation	90 days
Muncharaz [50]	Undisclosed	65	Mixed ARF (CAP 63.1%, ARDS 18.5%)^c^	0 (0)	62^b^	97	36	44	Non-invasive ventilation (*N* = 34)	Face mask, pressure support	Invasive ventilation, tidal volume 8–10 ml/kg (PBW), Ppl < 35 (*N* = 31)	Mortality	Hospital discharge
Azoulay [8]	French Ministry of Health	776	Mixed ARF (Pneumonia 53.0%)^c^	776 (100)	64	132	33	NA	High-flow nasal oxygen (*N* = 388)	–	Standard oxygen (*N* = 388)	Mortality, intubation	90 days
He [39]	National Natural Science Foundation of China	200	ARDS due to CAP	19 (9.5)	55	231	25	34	Non-invasive ventilation (*N* = 102)	Face mask, pressure support	Standard oxygen (*N* = 98)	Mortality, intubation	Hospital discharge
Andino [47]	Spanish Ministry of Health, Social Services, and Equality	46	Mixed ARF (CAP 30%, HAP 26%)^c^	NA	60	96	32	34.3	High-flow nasal oxygen (N = 24)		Standard oxygen (N = 22)	Mortality, intubation	Hospital discharge
Awadallah [51]	None	52	ARDS	NA	52	94.5	NA	33	Non-invasive ventilation (*N* = 26)	Face mask, pressure support	Invasive ventilation, tidal volume 6–7 ml/kg (PBW), Ppl < 30 (*N* = 26)	Mortality	Hospital discharge
Grieco [53]	2017 Merck Sharp & Dohme SRL award	109	ARF in COVID-19 patients^c^	8 (7.3)	65^b^	102^b^	28^b^	34^b^	Non-invasive ventilation (*N* = 54)	Helmet, pressure support	High-flow nasal oxygen (*N* = 55)	Mortality, intubation	60 days
AlptekİnoĞlu Mendİl [48]	None	100	Mixed ARF (pneumonia 74%)^c^	100 (100)	59^b^	262^b^	NA	30^b^	High-flow nasal oxygen (*N* = 51)		Standard oxygen (*N* = 49)	Mortality, intubation	28 days

ALI, acute lung injury; ARDS, acute respiratory distress syndrome; ARF, acute respiratory failure; CAP, community-acquired pneumonia; CPAP, continuous positive airway pressure; COPD, chronic obstructive pulmonary disease; COVID-19, coronavirus disease 2019; CPO, cardiopulmonary oedema; HAP, hospital-acquired pneumonia; ICU, intensive care unit; NA, not available; NIV, non-invasive ventilation; P/F ratio, ratio of arterial oxygen partial pressure to fractional inspired oxygen; PaCO_2_, partial pressure of arterial carbon dioxide; Ppl, plateau pressure; PBW, predicted body weight; RR, respiratory rate

^a^Forty-two patients had a cardiac disease

^b^The measurement value was reported as median

^c^Excluded patients with CPO

### Study characteristics and risk of bias assessment

The participants, interventions, comparisons, outcomes, and cohort characteristics of the included trials are shown in Table 1 and Additional file 1: Table S3. The mean age at randomisation ranged from 46 to 73 years, the mean P/F ratio was predominantly < 200 (16 trials [64.0%]) [7, 8, 32, 34, 35, 38, 40, 41, 44, 45, 47, 49–53], and the mean partial pressure of arterial carbon dioxide (PaCO_2_) was > 50 mmHg in one trial (4.0%) [41]. Nine trials (36.0%) included immunocompromised patients [8, 34, 36–38, 43–45, 48]. Community-acquired pneumonia was the most common cause of AHRF in 14 trials (56.0%) [7, 8, 31, 32, 34–36, 38–40, 42, 44, 47, 50]. Helmet interfaces were used in three of five trials comparing CPAP with SOT [42–44] and in a trial comparing PSV with HFNO [53]. In two of three trials comparing PSV with IMV [49, 50], target tidal volume was set at 8 ml/kg (of the predicted body weight) or more for mechanically ventilated patients.

### Non-invasive respiratory management strategies and risk of short-term mortality

Twenty-three trials (3169 patients) were included in the short-term mortality analysis. The pair-wise comparisons are shown in Additional file 1: Figure S1. The risk of bias was determined to be high for the outcome of mortality in six (26.1%) trials (Additional file 1: Table S4). We did not rate down due to publication bias (the funnel plot shown in Additional file 1: Figure S2); however, we assessed that the risk of bias was substantial between CPAP and SOT and; therefore, rated down. We also rated down considering inconsistencies in the direct comparisons of CPAP versus SOT, PSV versus IMV, and PSV versus HFNO (Additional file 1: Table S5). Incoherence between the direct and indirect RRs was observed for the comparisons of HFNO versus SOT, PSV versus SOT, and PSV versus HFNO. We also identified a significant global incoherence across the network.

**Fig. 3:**
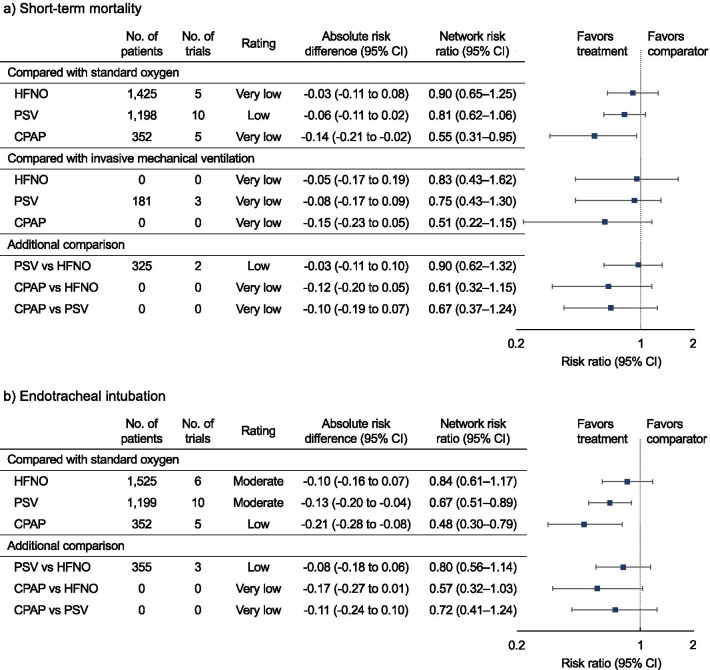
Forest plots for association of non-invasive respiratory management strategies with study outcomes. **a** For the primary outcome, short-term mortality, the longest follow-up was up to 100 days. **b** Secondary outcome, endotracheal intubation. All outcomes are reported as network risk ratios and absolute risk differences with 95% CIs. For estimating risk ratios for the comparison of HFNO vs IMV, CPAP vs IMV, CPAP vs HFNO, and CPAP vs PSV, only indirect evidence was used, because no direct pair-wise comparisons were available. The estimated absolute risks of mortality and endotracheal intubation were 30% and 40%, respectively, in the control group. CI, confidence interval; CPAP, continuous positive airway pressure; HFNO, high-flow nasal oxygen; IMV, invasive mechanical ventilation; PSV, pressure support ventilation; RR, risk ratio; SOT, standard oxygen therapy

Using SOT as the reference, CPAP (RR 0.55 [95% CI 0.31–0.95]; risk difference [RD] − 0.14 [95% CI − 0.21 to − 0.02]; very low certainty) was associated significantly with a lower risk of mortality (Fig. 3). Compared with SOT, PSV (RR 0.81 [95% CI 0.62–1.06]; RD, − 0.06 [95% CI − 0.11 to 0.02]; low certainty) and HFNO (RR, 0.90 [95% CI 0.65–1.25]; RD − 0.03 [95% CI − 0.11 to 0.08]; very low certainty) were not associated with a statistically significant lower risk of mortality.

Compared with IMV, CPAP (RR 0.51 [95% CI 0.22–1.15]; RD − 0.15 [95% CI − 0.23 to 0.05]; very low certainty), PSV (RR 0.75 [95% CI 0.43–1.30]; RD − 0.08 [95% CI − 0.17 to 0.09]; very low certainty), and HFNO (RR 0.83 [95% CI 0.43–1.62]; RD − 0.05 [95% CI − 0.17 to 0.19]; very low certainty) were not associated with a statistically significant lower risk of mortality, and all certainties of evidence were very low. Although CPAP tended to be associated with a lower risk of mortality, there were no significant differences among the non-invasive respiratory management strategies. The probability of being the best in reducing short-term mortality among all possible interventions was higher for CPAP, followed by PSV and HFNO; IMV and SOT tied for the worst (Table 2; Additional file 1: Figure S3).

**Table 2:** Results of network rank test in the Network Meta-analysis

	CPAP	PSV	HFNO	IMV	SOT
*a. Short-term mortality*					
Best	84.0%	8.1%	3.8%	4.0%	0.1%
2nd	9.0%	52.6%	23.3%	12.1%	3.0%
3rd	3.9%	31.4%	32.1%	13.6%	19.0%
4th	1.9%	7.1%	26.9%	16.0%	48.1%
Worst	1.2%	0.8%	13.9%	54.3%	29.8%
Mean rank	1.3	2.4	3.2	4.0	4.0
SUCRA	93.2	65.0	44.1	23.9	23.9

	CPAP	PSV	HFNO	SOT
*b. Endotracheal intubation*				
Best	88.5%	10.8%	0.7%	0.0%
2nd	8.8%	79.0%	12.1%	0.1%
3rd	2.6%	10.0%	74.2%	13.2%
Worst	0.1%	0.2%	13.0%	86.7%
Mean rank	1.1	2.0	3.0	3.9
SUCRA	95.2	66.8	33.5	4.5

CPAP, continuous positive airway pressure; HFNO, high-flow nasal oxygenation; IMV, invasive mechanical ventilation; PSV, pressure support ventilation; SCURA, surface under the cumulative ranking; SOT, standard oxygen therapy

^a^For the primary outcome, short-term mortality, the longest follow-up was up to 100 days

^b^For the secondary outcome, endotracheal intubation

### Non-invasive respiratory management strategies and risk of endotracheal intubation

Twenty-two trials (3,118 patients) were included in the intubation analysis. Pair-wise comparisons are shown in Additional file 1: Figure S1. The risk of bias was determined to be high for the outcome of intubation in six (27.3%) trials (Additional file 1: Table S4). We assessed that the risk of bias was serious between CPAP and SOT and; therefore, rated down. We did not rate down due to publication bias (funnel plot shown in Additional file 1: Figure S2) and incoherence. We rated down because serious inconsistencies were observed in the comparisons of PSV vs SOT and CPAP vs SOT (Additional file 1: Table S5).

Using SOT as the reference, CPAP (RR 0.48 [95% CI 0.30–0.79]; RD − 0.21 [95% CI − 0.28 to − 0.08]; low certainty) and PSV (RR 0.67 [95% CI 0.51–0.89]; RD − 0.13 [95% CI − 0.20 to − 0.04]; moderate certainty) were associated with a lower risk of endotracheal intubation (Fig. 3). Compared with SOT, HFNO (RR, 0.84 [95% CI 0.61–1.17]; RD − 0.10 [95% CI − 0.16 to 0.07]; moderate certainty) was not associated with a statistically significant lower risk of endotracheal intubation. There were no significant differences in the additional comparisons. The probability of being the best in reducing endotracheal intubation among all the possible interventions was higher for CPAP, followed by PSV, HFNO, and SOT (Table 2; Additional file 1: Figure S3).

### Results of additional analyses

The results of a pre-planned sensitivity analysis excluding four studies using helmet interfaces revealed that CPAP was not associated with a lower mortality and incidence of intubation (Additional file 1: Table S6 and S7). However, for the studies comparing CPAP with SOT, which were included this analysis, there was concern with respect to the risk of bias [40, 41].

The results of the post hoc sensitivity analyses are shown in Additional file 1: Table S8. The observed association between CPAP and reduced risk of mortality remained significant when considering studies that included only patients with mild hypoxaemic respiratory failure (mean P/F ratio > 150), and immunocompromised patients, after excluding studies with high risk of bias. On the other hand, CPAP did not show significant efficacy compared with SOT, when considering studies that included only patients with severe hypoxaemic respiratory failure (mean P/F ratio ≤ 150), and after excluding studies that enrolled any patients with COPD, cardiopulmonary oedema, or with immunocompromised status. The association of HFNO and PSV with a lower risk of mortality was not significant across almost all of the sensitivity analyses.

## Discussion

### Summary of evidence

In the current network meta-analyses of trials of adults with AHRF, compared with SOT, CPAP decreased the risk of death and both CPAP and PSV were associated with a lower risk of endotracheal intubation. Meanwhile, the treatment effects were not different between non-invasive respiratory management strategies and IMV for mortality. Ranking analyses showed that CPAP was the best strategy for reducing mortality and intubation. As per the results of the sensitivity analyses for mortality, CPAP also showed significant efficacy in only a few analyses, whereas compared with SOT, PSV and HFNO were not effective in almost all of the analyses.

### Association with previous studies

Non-invasive ventilation is associated with a lower mortality in patients with acute respiratory failure due to cardiopulmonary oedema and COPD [2, 54]. However, the efficacy of non-invasive respiratory management strategies in patients with de novo AHRF has been unclear [6–8]. Liu et al. [55] performed a pair-wise meta-analysis to compare the use of helmet non-invasive ventilation with control strategies, including the use of face mask non-invasive ventilation and SOT, and demonstrated that helmet non-invasive ventilation was associated with reduced hospital mortality and intubation requirement. Although both CPAP and PSV showed significant benefit in the subgroup analyses, six of eight studies using PSV were conducted among patients with acute exacerbation of COPD. The efficacy of non-invasive ventilation according to ventilation modes could not be evaluated in patients with AHRF. In 2020, Ferreyro et al. [10] reported an NMA in which the efficacy of non-invasive respiratory management strategies were compared with that of SOT among adult patients with AHRF and found that helmet non-invasive ventilation was associated with a lower risk of mortality and intubation compared with SOT, HFNO, and face mask non-invasive ventilation. However, the NMA included patients with postoperative respiratory failure or chest trauma. Those patients had various causes of respiratory failure, including atelectasis due to poor pain control, chest wall injury, and pleural effusion, not only because of lung injury. We included trials in which more than half of the patients were experiencing de novo AHRF. Although the cause of AHRF was still inconsistent, our analysis included a higher proportion of patients with de novo AHRF compared with the previous NMA.

There were insufficient data examining HFNO compared to non-invasive ventilation among patients with de novo AHRF. As per the results from an RCT comparing the use of helmet PSV with HFNO in patients with AHRF due to the coronavirus disease 2019, helmet PSV was associated with a higher P/F ratio and PaCO_2_ [53]. Although the rate of intubation was lower in patients with the use of a helmet PSV, the mortality was not different. A helmet interface can decrease air leaks and provide higher levels of PEEP, potentially increasing alveolar recruitment and improving oxygenation [56], but increasing dead space may worsen ventilation and contribute to lager tidal volume. Although the PEEP effect of HFNO may not be sufficient to avoid intubation, it is unclear which is a better strategy, HFNO or non-invasive ventilation, considering dead space. In our NMA, HFNO did not show a reduction in the rate of mortality and incidence of intubation compared with other respiratory management strategies. Further evaluation is needed to provide conclusive recommendations, although HFNO was recommended for patients with AHRF compared with SOT [3].

In all trials comparing helmet non-invasive ventilation with SOT, which were included in the previous NMA, CPAP was used as a ventilation mode [10]. The use of CPAP might contribute to the superiority of helmet non-invasive ventilation. According to an RCT comparing helmet interface to face mask in patients who underwent non-invasive ventilation, patients with a helmet interface were set at a lower level of pressure support and had a lower mortality [57]. Since excessive tidal volume may worsen outcomes [9], it may be important to set lower levels of pressure support for patients with AHRF. CPAP also has advantages over non-invasive ventilation in terms of having simpler technology, better synchrony, and requiring potentially less expensive equipment [6]. Our findings imply that CPAP is the most effective among the non-invasive respiratory management strategies, in concordance with these physiological effects.

### Significance and implications

A high respiratory drive and large tidal volume may contribute to patient self-inflicted lung injury and poor outcomes in patients with AHRF [58–60]. In our NMA, PSV was not associated with lower mortality, but CPAP decreased mortality and the incidence of endotracheal intubation compared with SOT. Furthermore, ranking analyses showed that CPAP was the best strategy for reducing mortality and intubation. Normally, when CPAP is used as a primary ventilation mode, we do not use pressure support unless pressure support is needed (e.g. in patients with hypercapnia, those with a lack of tidal volume, and those with a high respiratory drive). It may contribute the reduction of using unnecessary pressure support. When performing non-invasive ventilation in patients with AHRF, PEEP recruiting the lungs and maintaining them open may reduce respiratory drive and contribute to lung protection. Although pressure support is needed for some patients with AHRF, we should use pressure support with caution as this may lead to excessive tidal volume and lung injury. An ongoing RCT (jRCTs052180236) may provide further evidence to support these claims.

Although non-invasive ventilation is performed to avoid intubation, treatment failure has been reported to occur in 37.5% of patients with AHRF [4]. Furthermore, treatment failure has been associated with hospital mortality. De novo AHRF, including ARDS, was one of the risk factors for non-invasive ventilation failure [61]. Despite the high risk of treatment failure, no meta-analyses have been reported to compare non-invasive respiratory management strategies with IMV. We did not find significant differences between non-invasive respiratory management strategies and IMV, which was not considered as lung-protective ventilation in most of the included trials, in a decrease of mortality. It remains unclear whether it is better to ensure lung protection or avoid complications of endotracheal intubation. CPAP demonstrated the efficacy in the sensitivity analysis among patients with mild hypoxaemia, but not with severe hypoxaemia. Since lung-protective ventilation using neuromuscular blockers is recommended strongly in patients with severe hypoxaemia [62], our findings imply that non-invasive management strategies should not be performed in such patients.

### Strengths and limitations

To the best of our knowledge, no systematic reviews and meta-analyses have been performed to evaluate non-invasive ventilation according to ventilation modes and compare them with IMV in adults with AHRF. However, the current NMA also had several limitations. First, language restrictions may have contributed to the inclusion of an inadequate number of studies. However, we did not identify any trials in other languages being included in previous meta-analyses that had no language restrictions [10, 63]. Therefore, we believe that the language restriction had no effect. Second, we included studies with patients with cardiopulmonary oedema and COPD who were at a low risk of non-invasive ventilation failure. This may contribute to overestimating the treatment effect. The NMA assumption was that individual trials enrolled similar populations, and that the intervention protocol was similar across the different studies. We need to interpret results from the current network meta-analysis with caution because of a variety of causes leading to AHRF. Similar to the results from a post hoc analysis that excluded patients with cardiopulmonary oedema and COPD, we did not find any significant efficacy with the implementation of non-invasive respiratory management strategies. Our findings did not provide conclusive evidence among patients with de novo AHRF. Third, the effect of non-invasive ventilation may not have been consistent with respect to patient severity [4]. The mean P/F ratio in studies in which IMV was compared was lower than that in studies in which SOT was compared. The differences in the treatment effect may affect intransitivity and incoherence in an NMA. Fourth, there was a concern about the primary studies included in our review regarding the lack of blinding of the treatment groups. Although this was unlikely to bias the assessment of hard outcomes, it may have contributed to performance bias. Fifth, we did not observe a significant benefit with CPAP in only a few sensitivity analyses. In contrast, PSV and HFNO were not effective in almost all of the analyses compared with SOT. Further studies evaluating CPAP with more participants are needed to provide robust evidence because most trials had a small sample size. Sixth, network RR was only estimated by indirect evidence in some comparisons. Specifically, few studies compared non-invasive respiratory management strategies with IMV. Further studies are needed to provide a higher certainty of evidence. Seventh, most studies did not report on tidal volume with predicted body weight. Thus, it was unclear whether pressure support was associated with larger tidal volume. Finally, the fact that the included studies reported different follow-up times for all-cause mortality may have contributed to the heterogeneity. However, as per the results of the sensitivity analyses, the effect on mortality within 30 days was similar to that from the main analysis.

## Conclusions

The current network meta-analysis demonstrated that CPAP may be the most effective respiratory management strategy among patients with AHRF. Considering the low certainty of the current evidence, particularly compared with IMV, further studies are required to clarify whether non-invasive respiratory management strategies for de novo AHRF are effective or not. When performing non-invasive ventilation among patients with de novo AHRF, it is important to avoid excessive tidal volume and lung injury. Although pressure support is needed for some of these patients, it should be applied with caution because this may lead to excessive tidal volume and lung injury. If the risk of lung injury cannot be avoided, we should ensure lung-protective ventilation with endotracheal intubation, especially in patients with severe hypoxaemia.

## Supplementary Information

**Additional file 1.** **Table S1.** PRISMA NMA checklist. **Table S2.** Search strategy. **Table S3.** The proportion of patients with cause of respiratory failure or situation for exclusion criteria. **Table S4.** Summary of risk of bias of the studies included in the network meta-analysis. **Table S5.** Summary of network meta-analysis and GRADE assessment for the effects of non-invasive respiratory management strategies. **Table S6.** Pre-planned sensitivity analysis for the effect of non-invasive respiratory management strategies on outcomes in the network meta-analysis (excluding studies with helmet interfaces). **Table S7.** Pre-planned sensitivity analyses for the network rank test in the network meta-analysis (excluding studies with helmet interfaces). Table S8 Post-hoc sensitivity analyses for the effect of non-invasive respiratory management strategies on short-term mortality in the network meta-analysis. **Fig. S1.** Summary of random effects meta-analysis for direct comparisons of non-invasive respiratory management strategies for adults with AHRF (RevMan 5.3). **Fig. S2.** Comparison adjusted funnel plot for the network meta-analysis. **Fig. S3.** Results of ranking probability in the network meta-analysis.

## Data Availability

The datasets used and analysed during the current network meta-analysis are available from the corresponding author upon reasonable request.

## Abbreviations

AHRF: Acute hypoxemic respiratory failure
ALI: Acute lung injury
ARDS: Acute respiratory distress syndrome
ARF: Acute respiratory failure
CAP: Community-acquired pneumonia
CI: Confidence interval
COPD: Chronic obstructive pulmonary disease
COVID-19: Coronavirus disease 2019
CPAP: Continuous positive airway pressure
GRADE: Grades of Recommendation, Assessment, Development and Evaluation Working Group
HFNO: High-flow nasal oxygen
ICU: Intensive care unit
IMV: Invasive mechanical ventilation
NMA: Network meta-analysis
PSV: Pressure support ventilation
P/F ratio: Ratio of arterial oxygen partial pressure to fractional inspired oxygen
PaCO_2_: Partial pressure of arterial carbon dioxide
PaO_2_: Partial pressure of arterial oxygen
PBW: Predicted body weight
PEEP: Positive end-expiratory pressure
RCT: Randomized controlled trial
RevMan: Review manager
RR: Risk ratio
RD: Risk difference
SOT: Standard oxygen therapy
SUCRA: Surface under the cumulative ranking curve
