# Supplementary material for: Efficacy of non-invasive and invasive respiratory management strategies in adult patients with acute hypoxaemic respiratory failure: a systematic review and network meta-analysis
Source: Crit Care. 2021 Nov 29;25:414. doi: 10.1186/s13054-021-03835-8 (PMC8628281; doi:10.1186/s13054-021-03835-8)
Supplement: Supplementary file 1 — Additional file 1. Table S1. PRISMA NMA checklist. Table S2. Search strategy. Table S3. The proportion of patients with cause of respiratory failure or situation for exclusion criteria. Table S4. Summary of risk of bias of the studies included in the network meta-analysis. Table S5. Summary of network meta-analysis and GRADE assessment for the effects of non-invasive respiratory management strategies. Table S6. Pre-planned sensitivity analysis for the effect of non-invasive respiratory management strategies on outcomes in the network meta-analysis (excluding studies with helmet interfaces). Table S7. Pre-planned sensitivity analyses for the network rank test in the network meta-analysis (excluding studies with helmet interfaces). Table S8 Post-hoc sensitivity analyses for the effect of non-invasive respiratory management strategies on short-term mortality in the network meta-analysis. Fig. S1. Summary of random effects meta-analysis for direct comparisons of non-invasive respiratory management strategies for adults with AHRF (RevMan 5.3). Fig. S2. Comparison adjusted funnel plot for the network meta-analysis. Fig. S3. Results of ranking probability in the network meta-analysis. [file 13054_2021_3835_MOESM1_ESM.pdf]

Efficacy of non-invasive and invasive respiratory management strategies in adult patients with acute hypoxaemic respiratory failure: A systematic review and network meta-analysis

Table of Contents

Tables

Table S1 PRISMA NMA checklist
…………………….. 2–5

Table S2 Search strategy
…………………….. 6–9

Table S3 The proportion of patients with cause of respiratory failure or situation for exclusion criteria
………..……………. 10

Table S4 Summary of risk of bias of the studies included in the network meta-analysis
…………………. 11–12

Table S5 Summary of network meta-analysis and GRADE assessment for the effects of non-invasive respiratory management strategies
…………………. 13–14

Table S6 Pre-planned sensitivity analysis for the effect of non-invasive respiratory management strategies on outcomes in the network meta-analysis (excluding studies with helmet interfaces)
…………………. 15–16

Table S7 Pre-planned sensitivity analyses for the network rank test in the network meta-analysis (excluding studies with helmet interfaces)
……………………. 17

Table S8 Post-hoc sensitivity analyses for the effect of non-invasive respiratory management strategies on short-term mortality in the network meta-analysis
…………………. 18–20

Figures

Fig. S1 Summary of random effects meta-analysis for direct comparisons of non-invasive respiratory management strategies (RevMan 5.3)
…………………. 21–22

Fig. S2 Comparison adjusted funnel plot for the network meta-analysis
..……………………. 23

Fig. S3 Results of ranking probability in the network meta-analysis
..……………………. 24

Supplementary Table S1 PRISMA NMA Checklist of Items to Include When Reporting A Systematic Review Involving a Network Meta-analysis

Section/Topic	Item #	Checklist Item	Reported on Page #
**TITLE**			
Title	1	Identify the report as a systematic review *incorporating a network meta-analysis (or related form of meta-analysis).*	#1
**ABSTRACT**			
Structured summary	2	Provide a structured summary including, as applicable:<br>**Background:** main objectives<br>**Methods:** data sources; study eligibility criteria, participants, and interventions; study appraisal; and *synthesis methods, such as network meta-analysis.*<br>**Results:** number of studies and participants identified; summary estimates with corresponding confidence/credible intervals; *treatment rankings may also be discussed. Authors may choose to summarize pairwise comparisons against a chosen treatment included in their analyses for brevity.*<br>**Discussion/Conclusions:** limitations; conclusions and implications of findings.<br>**Other:** primary source of funding; systematic review registration number with registry name.	#2–3
**INTRODUCTION**			
Rationale	3	Describe the rationale for the review in the context of what is already known*, including mention of why a network meta-analysis has been conducted.*_	#4–6
Objectives	4	Provide an explicit statement of questions being addressed, with reference to participants, interventions, comparisons, outcomes, and study design (PICOS).	#4–6
**METHODS**			
Protocol and registration	5	Indicate whether a review protocol exists and if and where it can be accessed (e.g., Web address); and, if available, provide registration information, including registration number.	#6
Eligibility criteria	6	Specify study characteristics (e.g., PICOS, length of follow-up) and report characteristics (e.g., years considered, language, publication status) used as criteria for eligibility, giving rationale. *Clearly describe eligible treatments included in the treatment network, and*	#6–9

		*note whether any have been clustered or merged into the same node (with justification).*_	
Information sources	7	Describe all information sources (e.g., databases with dates of coverage, contact with study authors to identify additional studies) in the search and date last searched.	#9
Search	8	Present full electronic search strategy for at least one database, including any limits used, such that it could be repeated.	#9–10
Study selection	9	State the process for selecting studies (i.e., screening, eligibility, included in systematic review, and, if applicable, included in the meta-analysis).	#9
Data collection process	10	Describe method of data extraction from reports (e.g., piloted forms, independently, in duplicate) and any processes for obtaining and confirming data from investigators.	#10
Data items	11	List and define all variables for which data were sought (e.g., PICOS, funding sources) and any assumptions and simplifications made.	#11
**Geometry of the network**	**S1**	Describe methods used to explore the geometry of the treatment network under study and potential biases related to it. This should include how the evidence base has been graphically summarized for presentation, and what characteristics were compiled and used to describe the evidence base to readers.	#11
Risk of bias within individual studies	12	Describe methods used for assessing risk of bias of individual studies (including specification of whether this was done at the study or outcome level), and how this information is to be used in any data synthesis.	#11–12
Summary measures	13	State the principal summary measures (e.g., risk ratio, difference in means). *Also describe the use of additional summary measures assessed, such as treatment rankings and surface under the cumulative ranking curve (SUCRA) values, as well as modified approaches used to present summary findings from meta-analyses.*	#12–13
Planned methods of analysis	14	Describe the methods of handling data and combining results of studies for each network meta-analysis. This should include, but not be limited to:<br>• *Handling of multi-arm trials;*<br>• *Selection of variance structure;*<br>• *Selection of prior distributions in Bayesian analyses; and*<br>• *Assessment of model fit.*	#12–13
**Assessment of Inconsistency**	**S2**	Describe the statistical methods used to evaluate the agreement of direct and indirect evidence in the treatment network(s) studied. Describe efforts taken to address its presence when found.	#13–14

	Risk of bias across studies	15	Specify any assessment of risk of bias that may affect the cumulative evidence (e.g., publication bias, selective reporting within studies).	#14
	Additional analyses	16	Describe methods of additional analyses if done, indicating which were pre-specified. This may include, but not be limited to, the following:<br>• Sensitivity or subgroup analyses;<br>• Meta-regression analyses;<br>• *Alternative formulations of the treatment network; and*<br>• *Use of alternative prior distributions for Bayesian analyses (if applicable).*_	#15
**RESULTS†**				
	Study selection	17	Give numbers of studies screened, assessed for eligibility, and included in the review, with reasons for exclusions at each stage, ideally with a flow diagram.	#15
	**Presentation of network structure**	**S3**	Provide a network graph of the included studies to enable visualization of the geometry of the treatment network.	#15–16
	**Summary of network geometry**	**S4**	Provide a brief overview of characteristics of the treatment network. This may include commentary on the abundance of trials and randomized patients for the different interventions and pairwise comparisons in the network, gaps of evidence in the treatment network, and potential biases reflected by the network structure.	#15–16
	Study characteristics	18	For each study, present characteristics for which data were extracted (e.g., study size, PICOS, follow-up period) and provide the citations.	#16
	Risk of bias within studies	19	Present data on risk of bias of each study and, if available, any outcome level assessment.	#16-20
	Results of individual studies	20	For all outcomes considered (benefits or harms), present, for each study: 1) simple summary data for each intervention group, and 2) effect estimates and confidence intervals. *Modified approaches may be needed to deal with information from larger networks.*	#16-20
	Synthesis of results	21	Present results of each meta-analysis done, including confidence/credible intervals. *In larger networks, authors may focus on comparisons versus a particular comparator (e.g. placebo or standard care), with full findings presented in an appendix. League tables and forest plots may be considered to summarize pairwise comparisons.* If additional summary measures were explored (such as treatment rankings), these should also be	#16-20

			presented.	
	**Exploration for inconsistency**	**S5**	Describe results from investigations of inconsistency. This may include such information as measures of model fit to compare consistency and inconsistency models, *P* values from statistical tests, or summary of inconsistency estimates from different parts of the treatment network.	#16-20
	Risk of bias across studies	22	Present results of any assessment of risk of bias across studies for the evidence base being studied.	#16-20
	Results of additional analyses	23	Give results of additional analyses, if done (e.g., sensitivity or subgroup analyses, meta-regression analyses*, alternative network geometries studied, alternative choice of prior distributions for Bayesian analyses,* and so forth).	#20–21
**DISCUSSION**				
	Summary of evidence	24	Summarize the main findings, including the strength of evidence for each main outcome; consider their relevance to key groups (e.g., healthcare providers, users, and policy-makers).	#22
	Limitations	25	Discuss limitations at study and outcome level (e.g., risk of bias), and at review level (e.g., incomplete retrieval of identified research, reporting bias). *Comment on the validity of the assumptions, such as transitivity and consistency. Comment on any concerns regarding network geometry (e.g., avoidance of certain comparisons).*	#26-27
	Conclusions	26	Provide a general interpretation of the results in the context of other evidence, and implications for future research.	#28–29
**FUNDING**				
	Funding	27	Describe sources of funding for the systematic review and other support (e.g., supply of data); role of funders for the systematic review. This should also include information regarding whether funding has been received from manufacturers of treatments in the network and/or whether some of the authors are content experts with professional conflicts of interest that could affect use of treatments in the network.	#30

PICOS = population, intervention, comparators, outcomes, study design.

* Text in italics indicateS wording specific to reporting of network meta-analyses that has been added to guidance from the PRISMA statement.

† Authors may wish to plan for use of appendices to present all relevant information in full detail for items in this section.

Supplementary Table S2 Search strategy

a) PubMed search strategy (Performed on May 30, 2021)

Number	Searched for
#1	"Hypoxia"[mh] OR hypox*[tiab] OR "Respiratory Insufficiency"[mh] OR respiratory depression*[tiab] OR respiratory failure*[tiab] OR ventilatory depression*[tiab] OR respiratory insufficienc*[tiab] OR "Dyspnea"[mh] OR dyspnea*[tiab] OR "shortness of breath"[tiab] OR "Respiratory Distress Syndrome, Adult"[mh] OR acute respiratory distress[tiab] OR adult respiratory distress[tiab] OR respiratory distress syndrome*[tiab] OR AHRF[tiab] OR RDS[tiab] OR ARDS[tiab] OR "Acute Lung Injury"[mh] OR acute lung injur*[tiab] OR ALI[tiab]
#2	"Respiratory Distress Syndrome, Newborn"[mh]
#3	#1 or #2
#4	"Noninvasive Ventilation"[mh] OR noninvasive ventilation*[tiab] OR non invasive ventilation*[tiab] OR NIV[tiab] OR NPPV[tiab] OR NIPPV[tiab] OR noninvasive positive pressure ventilation*[tiab] OR noninvasive mechanical ventilation*[tiab] OR noninvasive pressure support ventilation*[tiab] OR "Continuous Positive Airway Pressure"[mh] OR continuous positive airway pressure*[tiab] OR bilevel positive airway pressure*[tiab] OR biphasic positive airway pressure*[tiab] OR BIPAP[tiab]
#5	"Oxygen Inhalation Therapy"[mh] OR HFNC[tiab] OR HHFNC[tiab] OR HHHFNC[tiab] OR HFNO[tiab] OR HFNT[tiab] OR HFNOT[tiab] OR HFO[tiab] OR HFOT[tiab] OR NHF[tiab] OR NHFC[tiab] OR NHFT[tiab] OR NHFO[tiab] OR NHFOT[tiab] OR high flow therap*[tiab] OR high flow oxygen[tiab] OR nasal high flow[tiab]
#6	#4 or #5
#7	("Randomized Controlled Trial"[pt] OR "Controlled Clinical Trial"[pt] OR "Clinical Trials as Topic"[mh] OR randomized[tiab] OR placebo[tiab] OR randomly[tiab] OR trial[tiab] OR groups[tiab]) NOT (Animals [mh] NOT Humans [mh])
#8	#3 and #6 and #7

Search terms included “pediatric” or “neonate,” because this systematic review was originally performed for clinical questions in the Japanese ARDS clinical practice guideline for adults and paediatrics. We excluded the studies among paediatric patients in the screening process.

Supplementary Table S2 Search strategy

b) CENTRAL search strategy (Performed on May 30, 2021)

Number	Searched for
#1	[mh Hypoxia] OR hypox*:ti,ab OR [mh "Respiratory Insufficiency"] OR "respiratory depression":ti,ab OR "respiratory failure":ti,ab OR "ventilatory depression":ti,ab OR "respiratory insufficiency":ti,ab OR [mh Dyspnea] OR dyspnea:ti,ab OR "shortness of breath":ti,ab OR [mh "Respiratory Distress Syndrome, Adult"] OR "acute respiratory distress":ti,ab OR "adult respiratory distress":ti,ab OR "respiratory distress syndrome":ti,ab OR AHRF:ti,ab OR RDS:ti,ab OR ARDS:ti,ab OR [mh "Acute Lung Injury"] OR "acute lung injury":ti,ab OR ALI:ti,ab
#2	[mh "Respiratory Distress Syndrome, Newborn"]
#3	#1 OR #2
#4	[mh "Noninvasive Ventilation"] OR "noninvasive ventilation":ti,ab OR "non invasive ventilation":ti,ab OR NIV:ti,ab OR NPPV:ti,ab OR NIPPV:ti,ab OR "noninvasive positive pressure ventilation":ti,ab OR "noninvasive mechanical ventilation":ti,ab OR "noninvasive pressure support ventilation":ti,ab OR [mh "Continuous Positive Airway Pressure"] OR "continuous positive airway pressure":ti,ab OR "bilevel positive airway pressure":ti,ab OR "biphasic positive airway pressure":ti,ab OR BIPAP:ti,ab
#5	[mh "Oxygen Inhalation Therapy"] OR HFNC:ti,ab OR HHFNC:ti,ab OR HHHFNC:ti,ab OR HFNO:ti,ab OR HFNT:ti,ab OR HFNOT:ti,ab OR HFO:ti,ab OR HFOT:ti,ab OR NHF:ti,ab OR NHFC:ti,ab OR NHFT:ti,ab OR NHFO:ti,ab OR NHFOT:ti,ab OR "high flow therapy":ti,ab OR "high flow oxygen":ti,ab OR "nasal high flow":ti,ab
#6	#4 OR #5
#7	#3 AND #6

Search terms included “pediatric” or “neonate,” because this systematic review was originally performed for clinical questions in the Japanese ARDS clinical practice guideline for adults and paediatrics. We excluded the studies among paediatric patients in the screening process.

Supplementary Table S2 Search strategy

c) Embase search strategy (Performed on May 30, 2021)

Number	Searched for
S1	(EMB.EXACT("hypoxia") OR (EMB.EXACT("paroxysmal dyspnea") OR EMB.EXACT("dyspnea")) OR (EMB.EXACT.EXPLODE("respiratory failure")))
S2	(EMB.EXACT.EXPLODE("neonatal respiratory distress syndrome") OR EMB.EXACT.EXPLODE("acute lung injury") OR EMB.EXACT.EXPLODE("adult respiratory distress syndrome"))
S3	(TI,AB(hypoxi* OR (respiratory p/0 (depression* OR failure* OR insufficienc*)) OR (ventilatory p/0 depression*) OR dyspnea* OR "shortness of breath" OR "acute respiratory distress" OR "adult respiratory distress" OR "respiratory distress syndrome" OR AHRF OR RDS OR ARDS OR (acute p/0 lung p/0 injur*) OR ALI))
S4	(S1 or S2 or S3)
S5	(EMB.EXACT("noninvasive ventilation"))
S6	(TI,AB("noninvasive ventilation" OR "non invasive ventilation" OR NIV OR NPPV OR NIPPV OR "noninvasive positive pressure ventilation" OR "noninvasive mechanical ventilation" OR "noninvasive pressure support ventilation" OR "continuous positive airway pressure" OR "bilevel positive airway pressure" OR "biphasic positive airway pressure" OR BIPAP))
S7	(EMB.EXACT("home oxygen therapy") OR EMB.EXACT("hyperbaric oxygen therapy") OR EMB.EXACT("oxygen therapy"))
S8	(TI,AB(HFNC OR HHFNC OR HHHFNC OR HFNO OR HFNT OR HFNOT OR HFO OR HFOT OR NHF OR NHFC OR NHFT OR NHFO OR NHFOT OR ("high flow" p/0 (therap* OR oxygen)) OR "nasal high flow"))
S9	(S5 or S6 or S7 or S8)
S10	((EMB.EXACT("controlled clinical trial") OR EMB.EXACT.EXPLODE("clinical trial (topic)") OR EMB.EXACT("randomized controlled trial")) OR (TI,AB(randomized) OR TI,AB(randomly) OR TI(trial) OR TI,AB(groups)) NOT (ANIMAL(YES) NOT HUMAN(YES)))
S11	(S4 and S9 and S10)

Search terms included “pediatric” or “neonate,” because this systematic review was originally performed for clinical questions in the Japanese ARDS clinical practice guideline for adults and paediatrics. We excluded the studies among paediatric patients in the screening process.

Supplementary Table S2 Search strategy

d) Ichushi search strategy (Performed on May 30, 2021)

Number	Searched for
#1	酸素欠乏/TH or 酸素欠乏/TA or anoxia/TA or Hypoxia/TA
#2	呼吸窮迫症候群-急性/TH or 急性呼吸窮迫症候群/TA or ARDS/TA
#3	急性肺損傷/TH or 急性肺損傷/TA or 急性肺障害/TA or 急性肺傷害/TA or ALI/TA
#4	呼吸不全/TH or 呼吸不全/TA or AHRF/TA
#5	呼吸困難/TH or 呼吸困難/TA
#6	呼吸窮迫症候群-新生児/TH or 新生児呼吸窮迫症候群/TA
#7	#1 or #2 or #3 or #4 or #5 or #6
#8	非侵襲的補助換気/TH or 非侵襲的補助換気/TA or NPPV/TA or NIPPV/TA
#9	持続気道陽圧/TH or 持続気道陽圧/TA or CPAP/TA
#10	非侵襲的陽圧呼吸/TH or 非侵襲的陽圧呼吸/TA or BIPAP/TA
#11	酸素吸入療法/TH or 酸素吸入/TA
#12	酸素療法/TA or ハイフロー/TA or HFNC/TA or NHF/TA or HFO/TA
#13	#8 or #9 or #10 or #11 or #12
#14	#7 and #13
#15	(#14) and (PT=会議録除く)
#16	ランダム化比較試験/TH or ランダム化/AL or 無作為化/AL
#17	比較試験/AL
#18	臨床試験/TH or 臨床試験/AL
#19	プラセボ/TH or プラセボ/AL
#20	対照/AL
#21	コントロール/AL
#22	臨床研究・疫学研究/TH or 臨床研究/AL
#23	#16 or #17 or #18 or #19 or #20 or #21 or #22
#24	#15 and #23

Search terms included "pediatric" or "neonate," because this systematic review was originally performed for clinical questions in the Japanese ARDS clinical practice guideline for adults and paediatrics. We excluded the studies among paediatric patients in the screening process.

Supplementary Table S3 The proportion of patients with cause of respiratory failure or situation for exclusion criteria

Source	CPO, %	COPD or asthma, %	Hypercapnia, %	Post-extubation, %	Post-surgery, %	Trauma, %	DNR, %
Wysock 1995	34.1	0	41.5	NA	29.3	4.9	NA
Antolnelli 1998	18.8	0	26.6	NA	29.7	12.5	NA
Confalonieri 1999	0	41.1	NA	0	0	0	NA
Antolnelli 2000	22.5	0	25.0	0	0	0	NA
Delcaux 2000	0	0	0	0	0	0	NA
Martin 2000	NA	37.7	31.1	NA	NA	NA	0
Hilbert 2001	NA	0	0	0	0	0	NA
Ferrer 2003	28.6	3.8	0	NA	3.8	16.2	NA
Cosentini 2010	0	21.3	NA	NA	NA	NA	NA
Squadrone 2010	0	0	0	0	0	0	NA
Wermke 2012	0	NA	NA	0	0	0	NA
Zhan 2012	0	NA	0	NA	NA	7.5	NA
Brambilila 2014	0	0	0	NA	NA	NA	NA
Azevedo 2015	43.3	NA	NA	NA	NA	NA	NA
Frat 2015	0	0	0	0	0	0	0
Lemiale (CC) 2015	7.0	11.0	0	0	0	0	NA
Lemiale (JAMA) 2015	0	8.0	0	0	0	0	0
Jones 2016	14.2	26.1	NA	0	0	NA	0
Muncharaz 2017	0	0	NA	0	13.8	15.4	0
Azoulay 2018	0	31.2	0	NA	0	0	3.6
He 2019	0	0	0	NA	NA	0	NA
Andino 2020	0	0	0	NA	10.9	0	NA
Awadallaha 2021	0	0	0	NA	NA	NA	0
Grieco 2021	0	0	0	0	0	0	0
Mendil 2021	0	NA	0	0	0	0	0

COPD, chronic obstructive pulmonary disease**;** CPO, cardiopulmonary oedema; DNR, do-not-resuscitate orders**.**

Supplementary Table S4 Summary of risk of bias of the studies included in the network meta-analysis

a) Short-term mortality

Source	Bias arising from the randomization process	Bias due to deviations from intended interventions	Bias due to missing outcome data	Bias in measurement of the outcome	Bias in selection of the reported result	Overall risk of bias
Wysock 1995	Low	Some concerns	Low	Low	Low	Some concerns
Antolnelli 1998	Some concerns	Some concerns	Low	Low	Low	High
Confalonieri 1999	Low	Some concerns	Low	Low	Low	Some concerns
Antonelli 2000	Low	Some concerns	Low	Low	Low	Some concerns
Delcaux 2000	Low	Some concerns	Some concerns	Low	Low	High
Martin 2000	Some concerns	Some concerns	Low	Low	Low	High
Hilbert 2001	Low	Some concerns	Low	Low	Low	Some concerns
Ferrer 2003	Low	Some concerns	Low	Low	Low	Some concerns
Cosentini 2010	Low	Some concerns	Low	Low	Low	Some concerns
Squadrone 2010	Low	Some concerns	Low	Low	Low	Some concerns
Wermke 2012	Low	Some concerns	Some concerns	Low	Low	High
Zhan 2012	Low	Some concerns	Low	Low	Low	Some concerns
Brambilila 2014	Some concerns	Some concerns	Low	Low	Low	High
Frat 2015	Low	Some concerns	Low	Low	Low	Some concerns
Lemiale (JAMA) 2015	Low	Some concerns	Low	Low	Low	Some concerns
Jones 2016	Low	Some concerns	Low	Low	Low	Some concerns
Muncharaz 2017	Low	Some concerns	Low	Low	Low	Some concerns
Azoulay 2018	Low	Some concerns	Low	Low	Low	Some concerns
He 2019	Low	Some concerns	Low	Low	Low	Some concerns
Andino 2020	Low	Some concerns	Low	Low	Low	Some concerns
Awadallaha 2021	Some concerns	Some concerns	Low	Low	Low	High
Grieco 2021	Low	Some concerns	Low	Low	Low	Some concerns
Mendil 2021	Some concerns	Some concerns	Low	Low	Low	High

Supplementary Table S4 Summary of risk of bias of the studies included in the network meta-analysis

b) Endotracheal intubation

Source	Bias arising from the randomization process	Bias due to deviations from intended interventions	Bias due to missing outcome data	Bias in measurement of the outcome	Bias in selection of the reported result	Overall risk of bias
Wysock 1995	Low	Some concerns	Low	Low	Low	Some concerns
Confalonieri 1999	Low	Some concerns	Low	Low	Low	Some concerns
Antonelli 2000	Low	Some concerns	Low	Low	Low	Some concerns
Delcaux 2000	Low	Some concerns	Some concerns	Low	Low	High
Martin 2000	Some concerns	Some concerns	Low	Low	Low	High
Hilbert 2001	Low	Some concerns	Low	Low	Low	Some concerns
Ferrer 2003	Low	Some concerns	Low	Low	Low	Some concerns
Cosentini 2010	Low	Some concerns	Low	Low	Low	Some concerns
Squadrone 2010	Low	Some concerns	Low	Low	Low	Some concerns
Wermke 2012	Low	Some concerns	Some concerns	Low	Low	High
Zhan 2012	Low	Some concerns	Low	Low	Low	Some concerns
Brambilila 2014	Some concerns	Some concerns	Low	Low	Low	High
Azevedo 2015	Some concerns	Some concerns	Low	Low	Low	High
Frat 2015	Low	Some concerns	Low	Low	Low	Some concerns
Lamiale (CC) 2015	Low	Some concerns	Low	Low	Low	Some concerns
Lemiale (JAMA) 2015	Low	Some concerns	Low	Low	Low	Some concerns
Jones 2016	Low	Some concerns	Low	Low	Low	Some concerns
Azoulay 2018	Low	Some concerns	Low	Low	Low	Some concerns
He 2019	Low	Some concerns	Low	Low	Low	Some concerns
Andino 2020	Low	Some concerns	Low	Low	Low	Some concerns
Grieco 2021	Low	Some concerns	Low	Low	Low	Some concerns
Mendil 2021	Some concerns	Some concerns	Low	Low	Low	High

Supplementary Table S5 Summary of network meta-analysis and GRADE assessment for the effects of non-invasive respiratory management strategies

a) Short-term mortality

Comparison	Direct estimate (95% CI)	Rating	Indirect estimate (95% CI)	Rating	node split analysis (*P* value)	Network estimate (95% CI)	Rating
HFNO vs SOT	1.00 (0.72–1.38)	⊕⊕⊕◯ Moderate a)	0.48 (0.21–1.10)	⊕⊕◯◯ Low b), c)	0.068	0.90 (0.65–1.25)	⊕◯◯◯ Very low a), b), d)
PSV vs SOT	0.76 (0.59–0.98)	⊕⊕⊕⊕ High	1.74 (0.75–4.04)	⊕⊕◯◯ Low a), b)	0.077	0.81 (0.62–1.06)	⊕⊕◯◯ Low a), d)
CPAP vs SOT	0.55 (0.31–0.95)	⊕◯◯◯ Very low a), b), c)	NA	-	-	0.55 (0.31–0.95)	⊕◯◯◯ Very low a), b), c)
IMV vs SOT	NA	-	1.08 (0.59–1.98)	⊕◯◯◯ Very low a), b), c)	-	1.08 (0.59–1.98)	⊕◯◯◯ Very low a), b), c), d)
HFNO vs IMV	NA	-	0.83 (0.43–1.62)	⊕◯◯◯ Very low a), b), c)	-	0.83 (0.43–1.62)	⊕◯◯◯ Very low a), b), c), d)
PSV vs IMV	0.75 (0.43–1.30)	⊕◯◯◯ Very low a), b), c)	NA	-	-	0.75 (0.43–1.30)	⊕◯◯◯ Very low a), b), c), d)
CPAP vs IMV	NA	-	0.51 (0.22–1.15)	⊕◯◯◯ Very low a), b), c)	-	0.51 (0.22–1.15)	⊕◯◯◯ Very low a), b), c), d), d)
PSV vs HFNO	1.65 (0.92–2.95)	⊕⊕◯◯ Low a), b)	0.63 (0.41–0.99)	⊕⊕⊕⊕ High	0.005	0.90 (0.62–1.32)	⊕⊕◯◯ Low a), d)
CPAP vs HFNO	NA	-	0.61 (0.32–1.15)	⊕⊕◯◯ Low a), b)	-	0.61 (0.32–1.15)	⊕◯◯◯ Very low a), b), d), d)
CPAP vs PSV	NA	-	0.67 (0.37–1.24)	⊕⊕◯◯ Low a), b)	-	0.67 (0.37–1.24)	⊕◯◯◯ Very low a), b), d), d)

a) Serious imprecision, b) Serious inconsistency, c) Serious risk of bias, d) Serious incoherence.

Testing for global incoherence P= 0.023.

CI, confidence interval; CPAP, continuous positive airway pressure; HFNO, high-flow nasal oxygenation; IMV, invasive mechanical ventilation; NA, not applicable; PSV, pressure support ventilation; SOT, standard oxygen therapy

Supplementary Table S5 Summary of network meta-analysis and GRADE assessment for the effects of non-invasive respiratory management strategies

b) Endotracheal intubation

Comparison	Direct estimate (95% CI)	Rating	Indirect estimate (95% CI)	Rating	node split analysis (*P* value)	Network estimate (95% CI)	Rating
HFNO vs SOT	0.87 (0.62–1.23)	⊕⊕◯◯ Low [a)]	0.76 (0.42–1.39)	⊕◯◯◯ Very low [a), b)]	0.902	0.84 (0.61–1.17)	⊕⊕⊕◯ Moderate [a)]
PSV vs SOT	0.68 (0.53–0.89)	⊕⊕⊕◯ Moderate [b)]	0.67 (0.34–1.32)	⊕◯◯◯ Very low [a), b)]	0.680	0.67 (0.51–0.89)	⊕⊕⊕◯ Moderate [b)]
CPAP vs SOT	0.48 (0.30–0.79)	⊕⊕◯◯ Low [b), c)]	NA	-	-	0.48 (0.30–0.79)	⊕⊕◯◯ Low [b), c)]
PSV vs HFNO	0.92 (0.60–1.39)	⊕◯◯◯ Very low [a), b)]	0.68 (0.41–1.11)	⊕⊕◯◯ Low [b), d)]	0.653	0.80 (0.56–1.14)	⊕⊕◯◯ Low [a), b)]
CPAP vs HFNO	NA	-	0.57 (0.32–1.03)	⊕◯◯◯ Very low [b), c), d)]	-	0.57 (0.32–1.03)	⊕◯◯◯ Very low [a), b), c)]
CPAP vs PSV	NA	-	0.72 (0.41–1.24)	⊕◯◯◯ Very low [a), b), c)]	-	0.72 (0.41–1.24)	⊕◯◯◯ Very low [a), b), c)]

a) Serious imprecision, b) Serious inconsistency, c) Serious risk of bias

Testing for global incoherence P= 0.496.

CI, confidence interval; CPAP, continuous positive airway pressure; HFNO, high-flow nasal oxygenation; IMV, invasive mechanical ventilation; NA, not applicable; PSV, pressure support ventilation; SOT, standard oxygen therapy

Supplementary Table S6 Pre-planned sensitivity analysis for the effect of non-invasive respiratory management strategies on outcomes in the network meta-analysis (excluding studies with helmet interfaces)

a) Short-term mortality

comparison	All patients-Main analysis			Excluding studies with helmet interface		
	No. of studies	RR (95% CI)	Rating	No. of studies	RR (95% CI)	Rating
HFNO vs SOT	5	0.90 (0.65–1.25)	Very low	5	0.93 (0.68–1.27)	Very low
PSV vs SOT	10	0.81 (0.62–1.06)	Low	10	0.80 (0.62–1.04)	Low
CPAP vs SOT	5	0.55 (0.31–0.95)	Very low	2	0.79 (0.43–1.48)	Very low
HFNO vs IMV	0	0.83 (0.43–1.62)	Very low	0	0.87 (0.46–1.67)	Very low
PSV vs IMV	3	0.75 (0.43–1.30)	Very low	3	0.76 (0.45–1.27)	Very low
CPAP vs IMV	0	0.51 (0.22–1.15)	Very low	0	0.75 (0.32–1.74)	Very low
PSV vs HFNO	2	0.90 (0.62–1.32)	Low	1	0.87 (0.59–1.27)	Very low
CPAP vs HFNO	0	0.61 (0.32–1.15)	Very low	0	0.86 (0.43–1.70)	Very low
CPAP vs PSV	0	0.67 (0.37–1.24)	Very low	0	0.99 (0.51–1.92)	Very low

Testing for global incoherence: Overall RCTs, P= 0.023; Excluding studies with helmet interface, P=0.006.

CI, confidence interval; CPAP, continuous positive airway pressure; HFNO, high-flow nasal oxygenation; IMV, invasive mechanical ventilation; PSV, pressure support ventilation; RR, risk ratio; SOT, standard oxygen therapy

Supplementary Table S6 Pre-planned sensitivity analysis for the effect of non-invasive respiratory management strategies on outcomes in the network meta-analysis (excluding studies with helmet interfaces)

b) Endotracheal intubation

comparison	All patients-Main analysis			Excluding studies with helmet interfaces		
	No. of studies	RR (95%CI)	Rating	No. of studies	RR (95%CI)	Rating
HFNO vs SOT	6	0.84 (0.61–1.17)	Moderate	6	0.80 (0.59–1.10)	Moderate
PSV vs SOT	10	0.67 (0.51–0.89)	Moderate	10	0.70 (0.54–0.91)	High
CPAP vs SOT	5	0.48 (0.30–0.79)	Low	2	0.67 (0.39–1.16)	Very low
PSV vs HFNO	3	0.80 (0.56–1.14)	Low	2	0.87 (0.61–1.25)	Low
CPAP vs HFNO	0	0.57 (0.32–1.03)	Very low	0	0.83 (0.45–1.56)	Very low
CPAP vs PSV	0	0.72 (0.41–1.24)	Very low	0	0.95 (0.52–1.74)	Very low

Testing for global incoherence: Overall RCTs, P= 0.496; Excluding studies with helmet interface, P=0.349.

CI, confidence interval; CPAP, continuous positive airway pressure; HFNO, high-flow nasal oxygenation; IMV, invasive mechanical ventilation; PSV, pressure support ventilation; RR, risk ratio; SOT, standard oxygen therapy

Supplementary Table S7 Pre-planned sensitivity analyses for the network rank test in the network meta-analysis (excluding studies with helmet interfaces)

a) Short-term mortality

	CPAP	PSV	HFNO	IMV	SOT
Best	47.8%	33.8%	9.9%	8.1%	0.4%
2nd	15.4%	43.4%	23.3%	11.8%	6.1%
3rd	11.4%	18.4%	28.0%	15.1%	27.1%
4th	13.5%	4.1%	24.2%	15.4%	42.8%
Worst	11.9%	0.3%	14.6%	49.6%	23.6%
Mean rank	2.3	1.9	3.1	3.9	3.8
SUCRA	68.4	76.6	47.4	28.4	29.2

b) Endotracheal intubation

	CPAP	PSV	HFNO	SOT
Best	53.7%	36.7%	9.5%	0.1%
2nd	19.3%	46.6%	33.5%	0.6%
3rd	18.2%	16.2%	50.2%	15.4%
Worst	8.8%	0.5%	6.8%	83.9%
Mean rank	1.8	1.8	2.5	3.8
SUCRA	72.6	73.2	48.6	5.6

CPAP, continuous positive airway pressure; HFNO, high-flow nasal oxygenation; IMV, invasive mechanical ventilation; PSV, pressure support ventilation; SCURA, surface under the cumulative ranking; SOT, standard oxygen therapy

Table S8 Post-hoc sensitivity analyses for the effect of non-invasive respiratory management strategies on short-term mortality in the network meta-analysis

comparison	Compared with SOT			Compared with IMV			PSV vs HFNO	CPAP vs HFNO	CPAP vs PSV
	HFNO	PSV	CPAP	HFNO	PSV	CPAP			
All patients-Main analysis, 23 RCTs									
No. of studies	5	10	5	0	3	0	2	0	0
RR (95% CI)	0.81 (0.52–1.26)	0.79 (0.59–1.05)	0.54 (0.30–0.95)	0.95 (0.42–2.16)	0.92 (0.48–1.78)	0.63 (0.25–1.56)	0.97 (0.59–1.60)	0.66 (0.33–1.35)	0.68 (0.36–1.29)
Rating	Very low	Low	Very low	Very low	Very low	Very low	Low	Very low	Very low
Patients with mild hypoxaemic respiratory failure (mean P/F ratio > 150), 10 RCTs (2 studies did not report P/F ratio)									
No. of studies	2	6	3	0	0	0	1	0	0
RR (95% CI)	0.74 (0.42–1.31)	0.91 (0.62–1.35)	0.33 (0.15–0.75)	NA	NA	NA	1.23 (0.67–2.26)	0.45 (0.17–1.21)	0.36 (0.15–0.90)
Rating	Low	Low	Low	NA	NA	NA	Low	Very low	Low
Patients with severe hypoxaemic respiratory failure (mean P/F ratio ≤ 150), 11 RCTs (2 studies did not report P/F ratio)									
No. of studies	2	3	2	0	3	0	1	0	0
RR (95% CI)	0.99 (0.65–1.50)	0.69 (0.48–0.99)	0.84 (0.42–1.66)	1.08 (0.53–2.21)	0.76 (0.46–1.26)	0.92 (0.37–2.31)	0.70 (0.42–1.17)	0.85 (0.38–1.90)	1.21 (0.57–2.58)
Rating	Moderate	Moderate	Low	Low	Low	Low	Moderate	Low	Low
Excluding studies that enrolled only immunocompromised patients, 16 RCTs									
No. of studies	3	6	4	0	3	0	2	0	0
RR (95% CI)	0.77 (0.45–1.33)	0.80 (0.51–1.25)	0.66 (0.33–1.33)	0.72 (0.30–1.69)	0.74 (0.39–1.40)	0.61 (0.22–1.74)	1.03 (0.58–1.83)	0.86 (0.35–2.06)	0.83 (0.37–1.89)
Rating	Low	Very low	Very low	Very low	Very low	Very low	Very low	Very low	Very low

Including studies that enrolled only immunocompromised patients, 7 RCTs									
No. of studies	2	4	1	0	0	0	0	0	0
RR (95% CI)	0.99 (0.86–1.14)	0.81 (0.66–0.98)	0.20 (0.07–0.59)	NA	NA	NA	0.81 (0.64–1.04)	0.20 (0.07–0.59)	0.25 (0.08–0.74)
Rating	Low	Low	Low	NA	NA	NA	Low	Low	Low
Excluding studies that enrolled any patients with chronic obstructive pulmonary disease or cardiopulmonary oedema, 12 RCTs									
No. of studies	2	5	3	0	2	0	2	0	0
RR (95% CI)	0.77 (0.38–1.59)	0.83 (0.48–1.45)	0.49 (0.21–1.14)	0.74 (0.22–2.44)	0.80 (0.31–2.06)	0.46 (0.12–1.85)	1.08 (0.52–2.26)	0.63 (0.21–1.91)	0.58 (0.22–1.58)
Rating	Low	Moderate	Low	Low	Low	Low	Low	Low	Low
Excluding studies with high risk of bias, 16 RCTs									
No. of studies	4	9	2	0	1	0	2	0	0
RR (95% CI)	0.84 (0.61–1.18)	0.79 (0.61–1.03)	0.23 (0.08–0.73)	1.51 (0.60–3.80)	1.42 (0.61–3.29)	0.42 (0.10–1.77)	0.94 (0.65–1.36)	0.28 (0.08–0.91)	0.30 (0.09–0.95)
Rating	Low	Low	Low	Very low	Low	Low	Low	Very low	Very low
For short-term mortality within 30 days, 22 RCTs									
No. of studies	5	9	5	0	3	0	2	0	0
RR (95% CI)	0.93 (0.65–1.33)	0.80 (0.59–1.08)	0.54 (0.31–0.95)	0.87 (0.43–1.76)	0.75 (0.43–1.32)	0.51 (0.22–1.19)	0.86 (0.56–1.32)	0.58 (0.30–1.14)	0.68 (0.36–1.28)
Rating	Moderate	Moderate	Low	Low	Low	Low	Low	Low	Low
Studies published after 2000, 17 RCTs									
No. of studies	5	7	3	0	2	0	2	0	0
RR (95% CI)	0.91 (0.67–1.25)	0.86 (0.65–1.14)	0.25 (0.10–0.65)	0.91 (0.41–2.02)	0.86 (0.43–1.73)	0.25 (0.08–0.84)	0.94 (0.65–1.37)	0.28 (0.10–0.74)	0.29 (0.11–0.78)
Rating	Low	Low	Low	Very low	Very low	Very low	Low	Low	Low

Testing for global incoherence: Overall RCTs, P= 0.023; Only patients with mild respiratory failure, P=0.012; Only patients with severe respiratory failure, P=0.701; Excluding studies that enrolled any patients with acute exacerbations of chronic obstructive pulmonary disease or cardiopulmonary edema, P= 0.533; Excluding studies that enrolled only immunocompromised patients, P= 0.049; Studies including only immunocompromised patients, NA; Excluding studies with high risk of bias, P= 0.009; For mortality within 30 days, P=0.054; Studies published after 2000, P=0.025.

CI, confidence interval; CPAP, continuous positive airway pressure; HFNO, high-flow nasal oxygenation; IMV, invasive mechanical ventilation; NA, not applicable; PSV, pressure support ventilation; RCT, randomised controlled trial; RR, risk ratio; SOT, standard oxygen therapy

Supplementary Fig. S1 Summary of random effects meta-analysis for direct comparisons of non-invasive respiratory management strategies (RevMan 5.3)

1. HFNO vs SOT

a) Short-term mortality

Study or Subgroup	HFNO Events	HFNO Total	SOT Events	SOT Total	Weight	Risk Ratio M-H, Random, 95% CI	Year
Frat 2015	13	106	22	94	11.4%	0.52 [0.28, 0.98]	2015
Jones 2016	35	165	24	138	17.8%	1.22 [0.76, 1.95]	2016
Azoulay 2018	182	388	187	388	49.5%	0.97 [0.84, 1.13]	2018
Andino 2020	6	24	4	22	4.0%	1.38 [0.45, 4.24]	2020
Mendil 2021	23	51	18	49	17.3%	1.23 [0.76, 1.98]	2021
**Total (95% CI)**		**734**		**691**	**100.0%**	**1.00 [0.79, 1.26]**	
Total events	259		255				

Heterogeneity: $Tau^2 = 0.02$; $Chi^2 = 5.88$, df = 4 (P = 0.21); $I^2 = 32\%$
Test for overall effect: Z = 0.03 (P = 0.98)

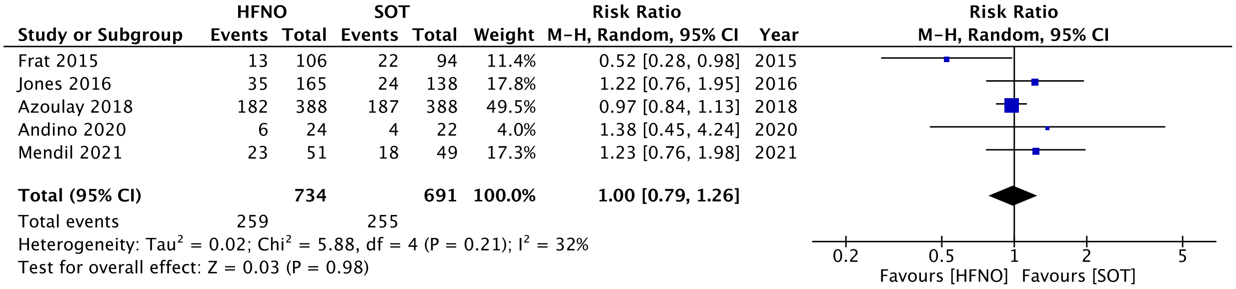

b) Endotracheal intubation

Study or Subgroup	HFNO Events	HFNO Total	SOT Events	SOT Total	Weight	Risk Ratio M-H, Random, 95% CI	Year
Frat 2015	40	106	44	94	28.9%	0.81 [0.58, 1.12]	2015
Lamiale (CC) 2015	4	52	2	48	2.3%	1.85 [0.35, 9.63]	2015
Jones 2016	1	165	3	138	1.3%	0.28 [0.03, 2.65]	2016
Azoulay 2018	150	388	170	388	44.1%	0.88 [0.75, 1.04]	2018
Andino 2020	8	24	14	22	12.1%	0.52 [0.27, 1.00]	2020
Mendil 2021	17	51	10	49	11.3%	1.63 [0.83, 3.21]	2021
**Total (95% CI)**		**786**		**739**	**100.0%**	**0.87 [0.67, 1.12]**	
Total events	220		243				

Heterogeneity: $Tau^2 = 0.03$; $Chi^2 = 7.73$, df = 5 (P = 0.17); $I^2 = 35\%$
Test for overall effect: Z = 1.09 (P = 0.28)

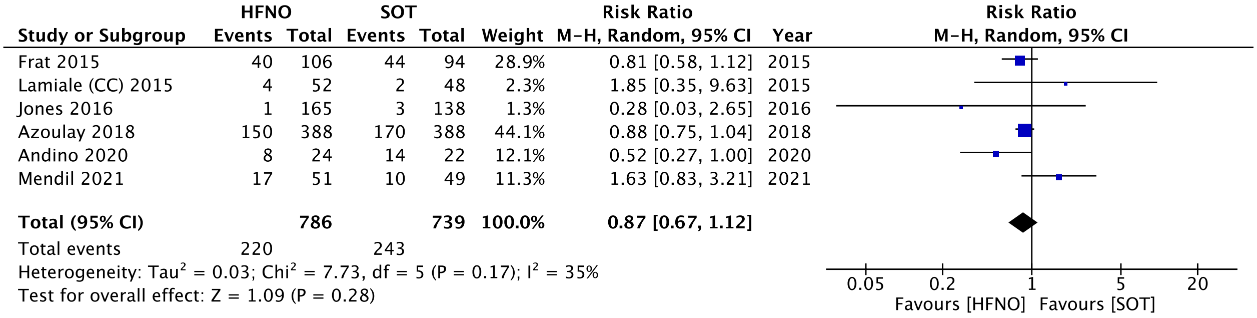

2. PSV vs SOT

a) Short-term mortality

Study or Subgroup	PSV Events	PSV Total	SOT Events	SOT Total	Weight	Risk Ratio M-H, Random, 95% CI	Year
Wysock 1995	7	21	10	20	5.8%	0.67 [0.32, 1.41]	1995
Confalonieri 1999	7	28	10	28	5.0%	0.70 [0.31, 1.58]	1999
Antonelli 2000	7	20	11	20	6.3%	0.64 [0.31, 1.30]	2000
Hilbert 2001	13	26	21	26	15.0%	0.62 [0.40, 0.95]	2001
Ferrer 2003	9	51	21	54	6.9%	0.45 [0.23, 0.90]	2003
Zhan 2012	1	21	5	19	0.8%	0.18 [0.02, 1.41]	2012
Wermke 2012	26	42	30	44	23.4%	0.91 [0.66, 1.24]	2012
Frat 2015	31	110	22	94	12.9%	1.20 [0.75, 1.93]	2015
Lemiale (JAMA) 2015	46	191	50	183	20.5%	0.88 [0.62, 1.25]	2015
He 2019	7	102	7	98	3.3%	0.96 [0.35, 2.64]	2019
**Total (95% CI)**		**612**		**586**	**100.0%**	**0.79 [0.65, 0.95]**	
Total events	154		187				

Heterogeneity: $Tau^2 = 0.01$; $Chi^2 = 10.73$, df = 9 (P = 0.29); $I^2 = 16\%$
Test for overall effect: Z = 2.45 (P = 0.01)

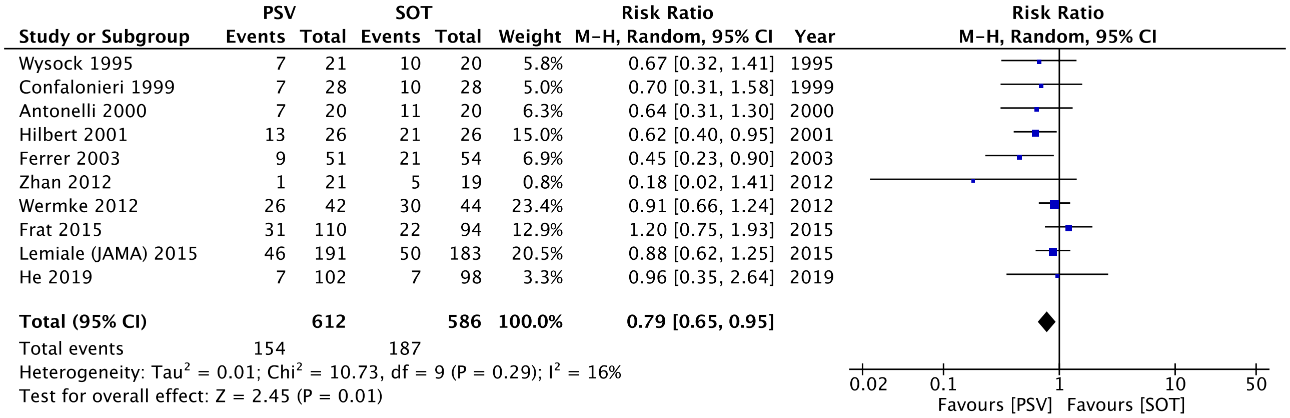

b) Endotracheal intubation

Study or Subgroup	PSV Events	PSV Total	SOT Events	SOT Total	Weight	Risk Ratio M-H, Random, 95% CI	Year
Wysock 1995	13	21	14	20	13.4%	0.88 [0.57, 1.38]	1995
Confalonieri 1999	6	28	17	28	7.6%	0.35 [0.16, 0.76]	1999
Antonelli 2000	4	20	14	20	6.0%	0.29 [0.11, 0.72]	2000
Hilbert 2001	12	26	20	26	12.9%	0.60 [0.38, 0.96]	2001
Ferrer 2003	13	51	28	54	11.4%	0.49 [0.29, 0.84]	2003
Wermke 2012	6	43	11	44	6.2%	0.56 [0.23, 1.37]	2012
Zhan 2012	1	21	4	19	1.5%	0.23 [0.03, 1.85]	2012
Frat 2015	55	110	44	94	17.1%	1.07 [0.80, 1.42]	2015
Lemiale (JAMA) 2015	73	191	82	183	18.2%	0.85 [0.67, 1.09]	2015
He 2019	9	102	7	98	5.7%	1.24 [0.48, 3.19]	2019
**Total (95% CI)**		**613**		**586**	**100.0%**	**0.68 [0.52, 0.89]**	
Total events	192		241				

Heterogeneity: $Tau^2 = 0.09$; $Chi^2 = 21.29$, df = 9 (P = 0.01); $I^2 = 58\%$
Test for overall effect: Z = 2.82 (P = 0.005)

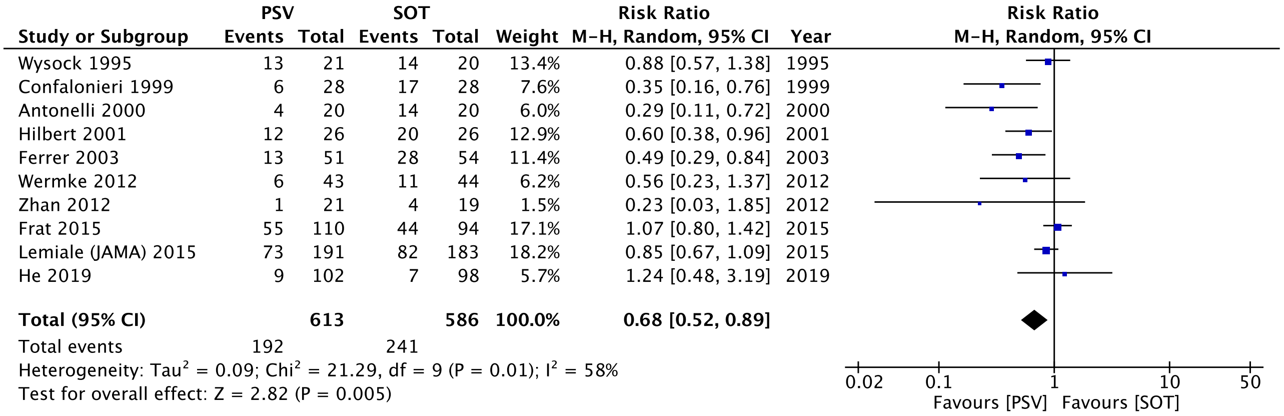

3. CPAP vs SOT

a) Short-term mortality

Study or Subgroup	CPAP Events	CPAP Total	SOT Events	SOT Total	Weight	Risk Ratio M-H, Random, 95% CI	Year
Delcaux 2000	19	61	18	61	33.0%	1.06 [0.62, 1.81]	2000
Martin 2000	5	32	10	29	25.9%	0.45 [0.18, 1.17]	2000
Squadrone 2010	3	20	15	20	23.7%	0.20 [0.07, 0.59]	2010
Cosentini 2010	0	20	0	27		Not estimable	2010
Brambilila 2014	2	40	7	41	17.4%	0.29 [0.06, 1.33]	2014
**Total (95% CI)**		**173**		**178**	**100.0%**	**0.46 [0.19, 1.07]**	
Total events	29		50				

Heterogeneity: $Tau^2 = 0.50$; $Chi^2 = 9.66$, df = 3 (P = 0.02); $I^2 = 69\%$

Test for overall effect: Z = 1.80 (P = 0.07)

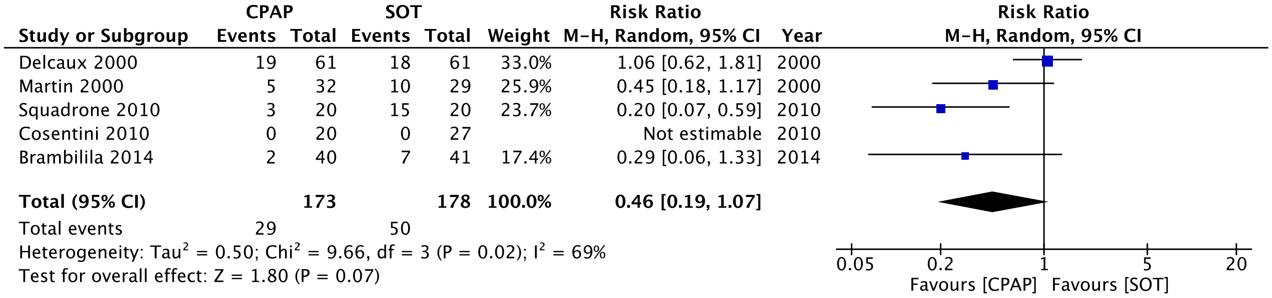

b) Endotracheal intubation

Study or Subgroup	CPAP Events	CPAP Total	SOT Events	SOT Total	Weight	Risk Ratio M-H, Random, 95% CI	Year
Delcaux 2000	21	62	24	61	32.3%	0.86 [0.54, 1.37]	2000
Martin 2000	9	32	17	29	28.5%	0.48 [0.25, 0.90]	2000
Cosentini 2010	0	20	0	27		Not estimable	2010
Squadrone 2010	2	20	8	20	13.9%	0.25 [0.06, 1.03]	2010
Brambilila 2014	6	40	26	41	25.3%	0.24 [0.11, 0.51]	2014
**Total (95% CI)**		**174**		**178**	**100.0%**	**0.44 [0.23, 0.86]**	
Total events	38		75				

Heterogeneity: $Tau^2 = 0.30$; $Chi^2 = 9.80$, df = 3 (P = 0.02); $I^2 = 69\%$

Test for overall effect: Z = 2.41 (P = 0.02)

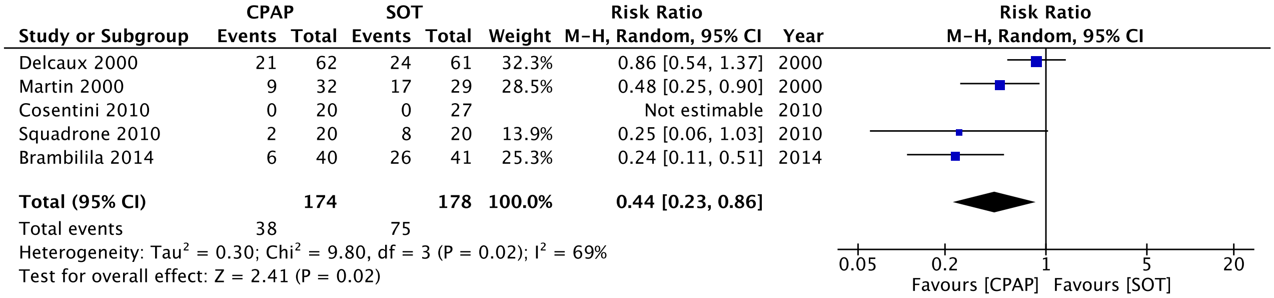

4. PSV vs IMV

a) Short-term mortality

Study or Subgroup	PSV Events	PSV Total	IMV Events	IMV Total	Weight	Risk Ratio M-H, Random, 95% CI	Year
Antolnelli 1998	10	32	16	32	37.9%	0.63 [0.34, 1.16]	1998
Muncharaz 2017	14	34	9	31	35.9%	1.42 [0.72, 2.80]	2017
Awadallah 2021	4	26	11	26	26.2%	0.36 [0.13, 1.00]	2021
**Total (95% CI)**		**92**		**89**	**100.0%**	**0.73 [0.35, 1.51]**	
Total events	28		36				

Heterogeneity: $Tau^2 = 0.27$; $Chi^2 = 5.67$, df = 2 (P = 0.06); $I^2 = 65\%$

Test for overall effect: Z = 0.85 (P = 0.39)

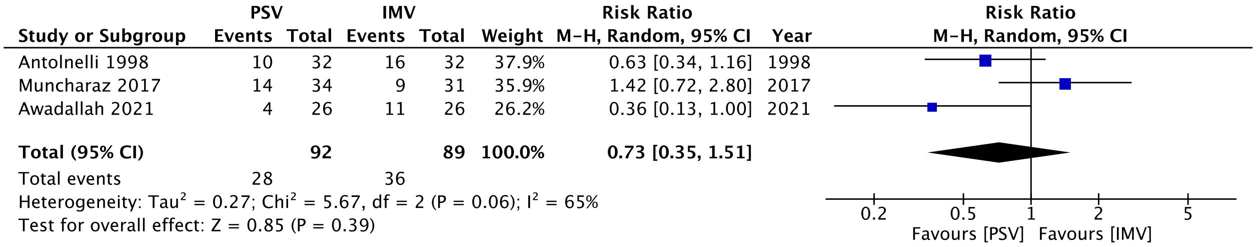

5. PSV vs HFNO

a) Short-term mortality

Study or Subgroup	PSV Events	PSV Total	HFNO Events	HFNO Total	Weight	Risk Ratio M-H, Random, 95% CI	Year
Frat 2015	31	110	13	106	53.0%	2.30 [1.27, 4.15]	2015
Grieco 2021	13	54	12	55	47.0%	1.10 [0.55, 2.20]	2021
**Total (95% CI)**		**164**		**161**	**100.0%**	**1.63 [0.79, 3.34]**	
Total events	44		25				

Heterogeneity: $Tau^2 = 0.16$; $Chi^2 = 2.53$, df = 1 (P = 0.11); $I^2 = 60\%$

Test for overall effect: Z = 1.33 (P = 0.18)

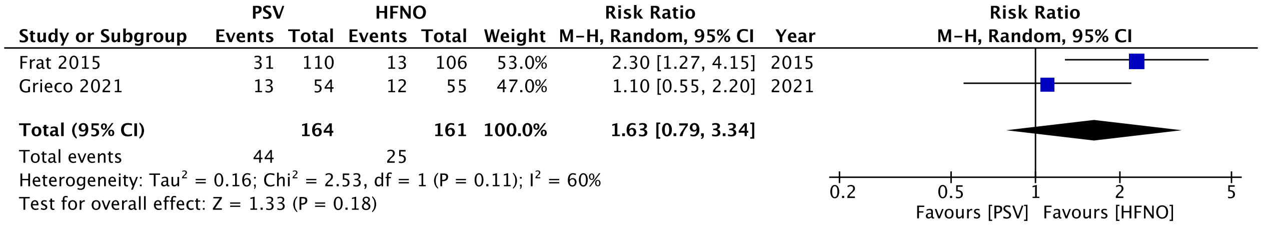

b) Endotracheal intubation

Study or Subgroup	PSV Events	PSV Total	HFNO Events	HFNO Total	Weight	Risk Ratio M-H, Random, 95% CI	Year
Azevedo 2015	9	16	9	14	28.9%	0.88 [0.49, 1.57]	2015
Frat 2015	55	110	40	106	38.8%	1.32 [0.97, 1.80]	2015
Grieco 2021	16	54	28	55	32.4%	0.58 [0.36, 0.95]	2021
**Total (95% CI)**		**180**		**175**	**100.0%**	**0.90 [0.53, 1.52]**	
Total events	80		77				

Heterogeneity: $Tau^2 = 0.16$; $Chi^2 = 8.17$, df = 2 (P = 0.02); $I^2 = 76\%$

Test for overall effect: Z = 0.39 (P = 0.70)

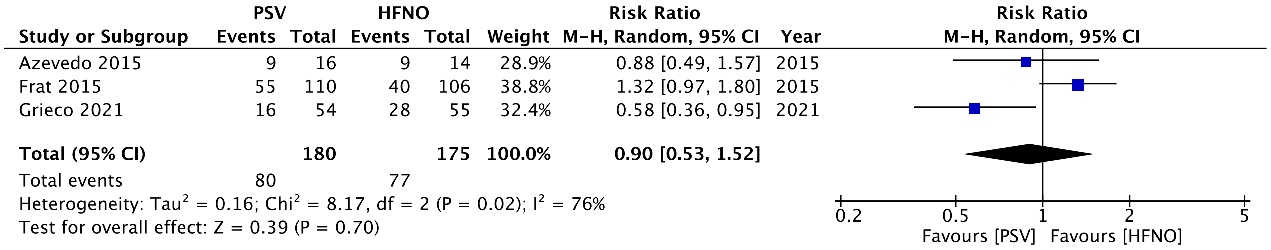

CI, confidence interval; CPAP, continuous positive airway pressure; HFNO, high-flow nasal oxygenation; IMV, invasive mechanical ventilation; PSV, pressure support ventilation; SOT, standard oxygen therapy

Supplementary Fig. S2 Comparison adjusted funnel plot for the network meta-analysis

a) Short-term mortality

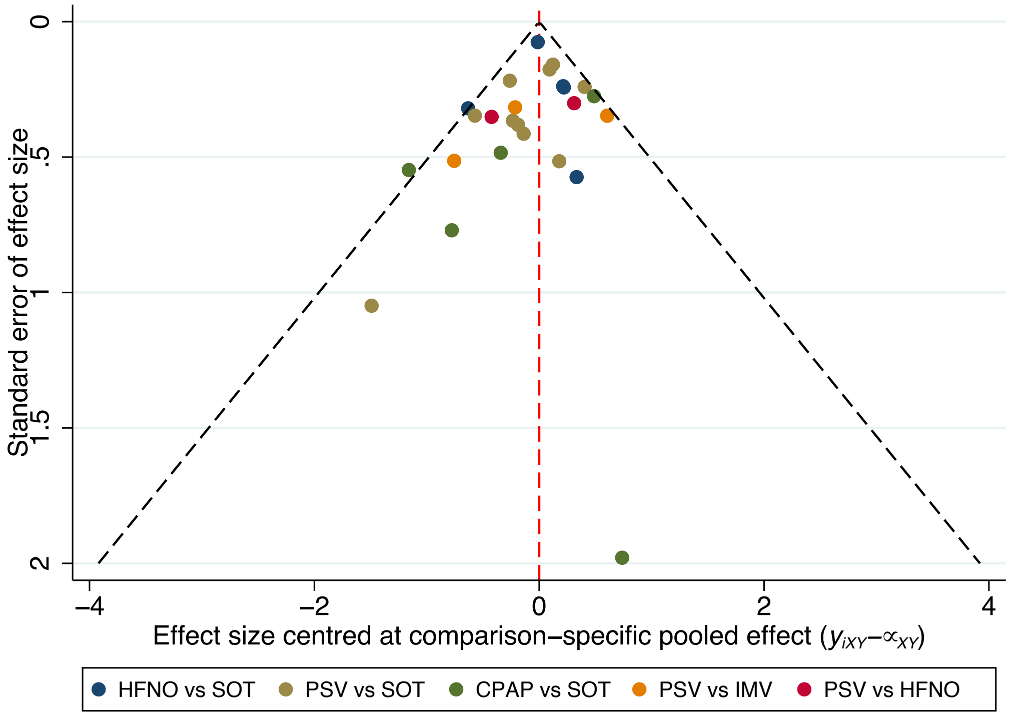

b) Endotracheal intubation

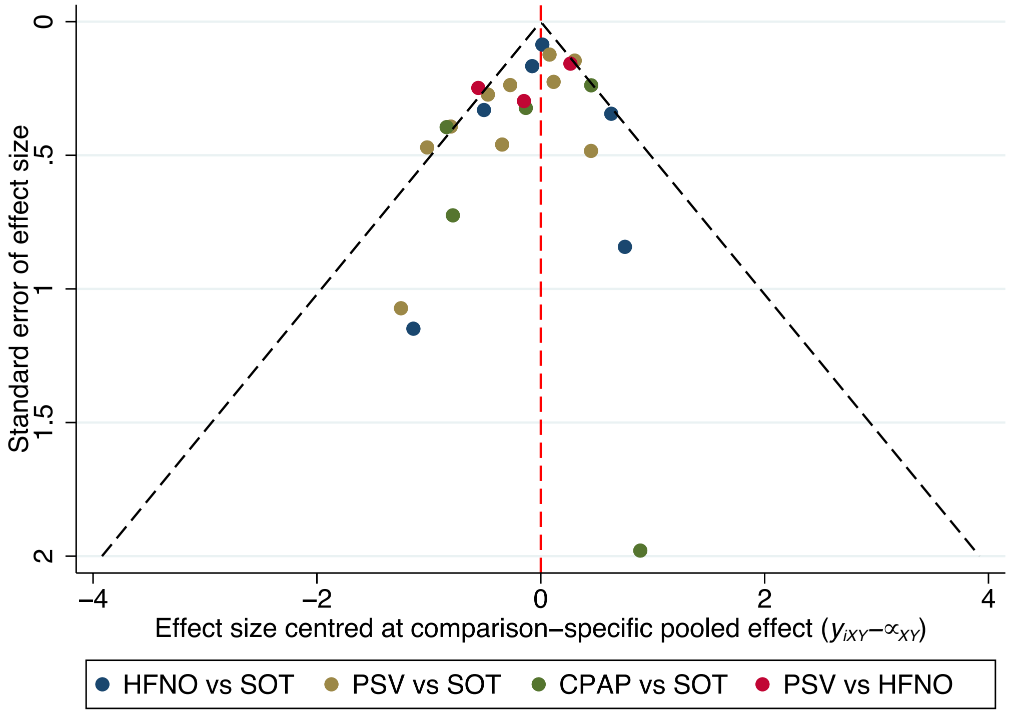

CPAP, continuous positive airway pressure; HFNO, high-flow nasal oxygenation; IMV, invasive mechanical ventilation; PSV, pressure support ventilation; SOT, standard oxygen therapy

Supplementary Fig. S3 Results of ranking probability in the network meta-analysis

a) Short-term mortality

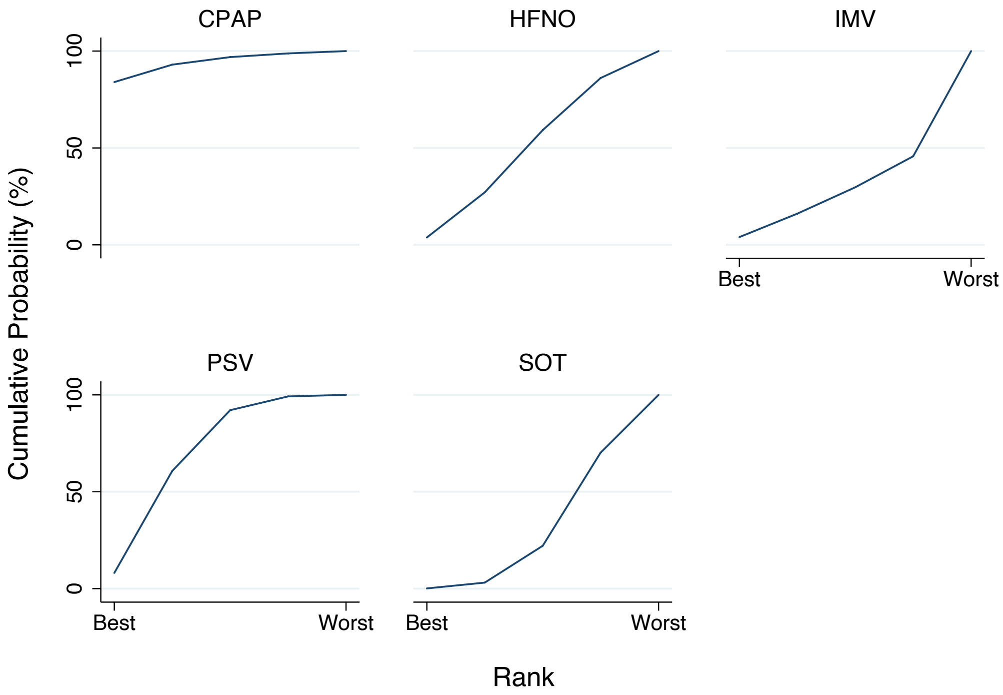

b) Endotracheal intubation

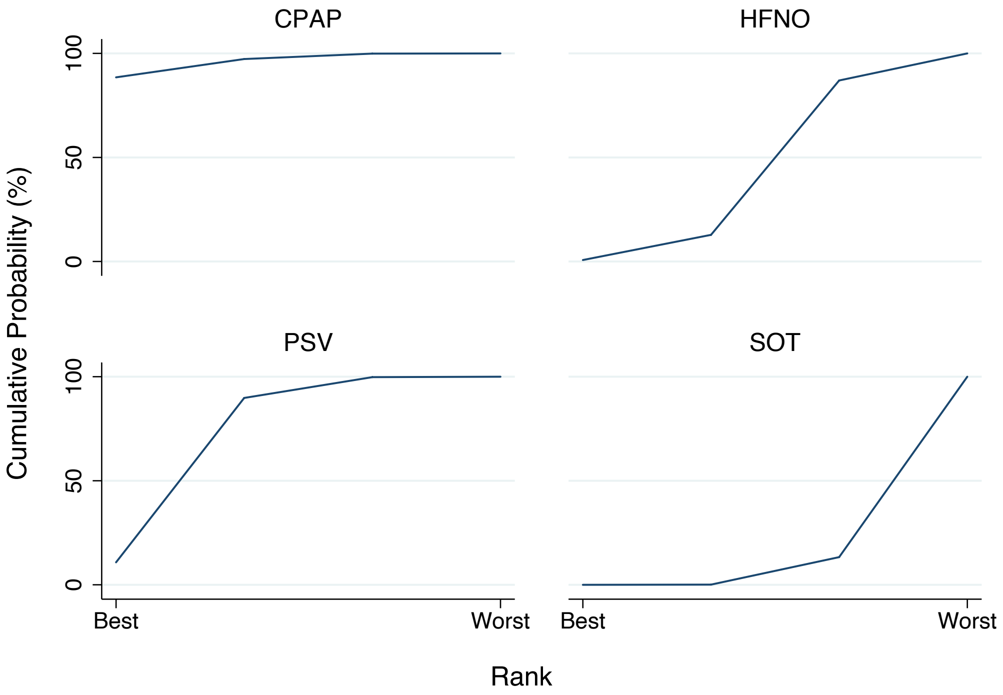

CPAP, continuous positive airway pressure; HFNO, high-flow nasal oxygenation; IMV, invasive mechanical ventilation; PSV, pressure support ventilation; SOT, standard oxygen therapy
